# Underground emissions and miners’ personal exposure to diesel and renewable diesel exhaust in a Swedish iron ore mine

**DOI:** 10.1007/s00420-022-01843-x

**Published:** 2022-03-16

**Authors:** Louise Gren, Annette M. Krais, Eva Assarsson, Karin Broberg, Malin Engfeldt, Christian Lindh, Bo Strandberg, Joakim Pagels, Maria Hedmer

**Affiliations:** 1 Ergonomics and Aerosol Technology, LTH, Lund University, 221 00 Lund, Sweden; 2 Division of Occupational and Environmental Medicine, Lund University, 221 00 Lund, Sweden; 3 Department of Occupational and Environmental Medicine, Region Skåne, 223 81 Lund, Sweden

**Keywords:** Underground mine, Occupational diesel exposure, PAHs, Biomarkers, Emission factors, Aftertreatment systems, Aerosols

## Abstract

**Purpose:**

Underground diesel exhaust exposure is an occupational health risk. It is not known how recent intensified emission legislation and use of renewable fuels have reduced or altered occupational exposures. We characterized these effects on multipollutant personal exposure to diesel exhaust and underground ambient air concentrations in an underground iron ore mine.

**Methods:**

Full-shift personal sampling (12 workers) of elemental carbon (EC), nitrogen dioxide (NO_2_), polycyclic aromatic hydrocarbons (PAHs), and equivalent black carbon (eBC) was performed. The study used and validated eBC as an online proxy for occupational exposure to EC. Ambient air sampling of these pollutants and particle number size distribution and concentration were performed in the vicinity of the workers. Urine samples (27 workers) were collected after 8 h exposure and analyzed for PAH metabolites and effect biomarkers (8-oxodG for DNA oxidative damage, 4-HNE-MA for lipid peroxidation, 3-HPMA for acrolein).

**Results:**

The personal exposures (geometric mean; GM) of the participating miners were 7 µg EC m^−3^ and 153 µg NO_2_ m^−3^, which are below the EU occupational exposure limits. However, exposures up to 94 µg EC m^−3^ and 1200 µg NO_2_ m^−3^ were observed. There was a tendency that the operators of vehicles complying with sharpened emission legislation had lower exposure of EC. eBC and NO_2_ correlated with EC, *R* = 0.94 and *R* = 0.66, respectively. No correlation was found between EC and the sum of 16 priority PAHs (GM 1790 ng m^−3^). Ratios between personal exposures and ambient concentrations were similar and close to 1 for EC and NO_2_, but significantly higher for PAHs. Semi-volatile PAHs may not be effectively reduced by the aftertreatment systems, and ambient area sampling did not predict the personal airborne PAHs exposure well, neither did the slightly elevated concentration of urinary PAH metabolites correlate with airborne PAH exposure.

**Conclusion:**

Miners’ exposures to EC and NO_2_ were lower than those in older studies indicating the effect of sharpened emission legislation and new technologies. Using modern vehicles with diesel particulate filter (DPF) may have contributed to the lower ambient underground PM concentration and exposures. The semi-volatile behavior of the PAHs might have led to inefficient removal in the engines aftertreatment systems and delayed removal by the workplace ventilation system due to partitioning to indoor surfaces. The results indicate that secondary emissions can be an important source of gaseous PAH exposure in the mine.

**Supplementary Information:**

The online version contains supplementary material available at 10.1007/s00420-022-01843-x.

## Introduction

Diesel exhaust is a known carcinogen (Group 1, IARC 2014) and a risk factor for lung cancer, as well as cardiovascular and respiratory diseases (Ilar et al. 2014; SBU (Swedish Council on Health Technology Asessment) 2017). Workers in underground mines are exposed to diesel exhaust emissions from mining equipment and other vehicles powered with diesel fuels. Still, there is limited knowledge about the levels and composition of diesel exposures in modern underground mines today. This is needed, for example, to create improved job-exposure matrices in epidemiological studies to allow future risk assessments (Plato et al. 2020; Audignon-Durand et al. 2021).

Diesel engine exhaust is a complex mixture containing both gas-phase and particle-phase emissions. Diesel exhaust particles generally consist of an elemental carbon (EC) core with a high surface area to which a large number of substances, such as metals and organics, can adsorb. The organic components include high molecular weight polycyclic aromatic hydrocarbons (PAHs), of which some are carcinogenic (Kim et al. 2013), and lubrication oil. In the gas phase, nitrogen oxides (NO_x_) that is nitric oxide (NO) and nitrogen dioxide (NO_2_), carbon monoxide (CO), and low molecular weight PAHs can be found (Matti Maricq 2007). European Union (EU) occupational exposure limits (OELs) of diesel exhaust have previously been limited to NO_2_ and carbon monoxide (CO), but from 2023 (2026 in underground mines) a new OEL of 50 µg EC m^−3^ will be implemented (Directive (EU) 2019/130 2019; Swedish Work Environment Authority 2020). A further reduction to as low as 10 µg EC m^−3^ has recently been implemented in a few European countries, namely Denmark and the Netherlands (Health Council of the Netherlands 2020; Arbejdstilsynet 2021).

EC is measured by time-integrated (offline) filter-based sampling to assess the full-shift average exposure concentration. EC can also be estimated as equivalent black carbon (eBC) (Cai et al. 2014; Yu et al. 2015) and measured with direct-reading instruments. This enables the identification of high-exposure work tasks (Lovén et al. 2020) that are not resolved with the time-integrated EC measurement. However, to the best of the authors’ knowledge, no studies of time-resolved personal exposure to eBC during underground mining have previously been published.

Occupational diesel exposures in mines have historically been high, and in literature before 2009, occupational diesel EC exposures have ranged from < 25 µg m^−3^ up to 660 µg m^−3^ (Pronk et al. 2009). Advances in emission legislation, engine development, and aftertreatment systems have reduced the hazardous exhaust emission from diesel vehicles, but breathing zone EC concentrations close to 100 µg m^−3^ have still been found in recent non-European mining studies (Debia et al. 2017; da Silveira Fleck et al. 2018).

Estimates of the lifetime mortality risk from lung cancer from 45 working years of occupational exposure to diesel (measured as EC) is 17 excess death per 10,000 for 1 µg EC m^−3^, and 200 excess deaths per 10,000 for 10 µg EC m^−3^ (Vermeulen et al. 2014). It should be noted that this does not include excess mortality risk from other diesel-related diseases, indicating that exposure levels need to be kept extremely low. Biomonitoring of diesel toxicity can include biomarkers related to oxidative damage: 4-hydroxynonenal mercapturic acid (4-HNE-MA) reflecting lipid peroxidation (Dalleau et al. 2013), 8-oxo-2′-deoxyguanosine (8-oxodG) reflecting DNA oxidative damage (Møller and Loft 2010), and 3-hydroxypropyl mercapturic acid (3-HPMA) which is a metabolite of acrolein (both from exposure and endogenously formed) reflecting oxidative stress and acrolein related diseases (Higashi et al. 2016). Thus, it is of key importance to monitor and understand how the emissions change with newer vehicles that comply with more stringent emission legislation, and how it changes effect biomarkers and exposure levels of EC, NO_2_, particle number (PN) and PAHs.

Emission legislation for heavy equipment (non-road engines) has become more stringent in recent years. It now includes more detailed limits for a range of components including PN and reduced NO_x_, hydrocarbon, CO, and particulate matter (PM) limits (Directive (EU) 2004/26/EC 2004; Regulation (EU) 2016/1628 2016). These reductions have been possible via technical development, such as the implementation of aftertreatment systems in the exhaust pipe. The ratios between different pollutants will also vary depending on engine operation mode (idling vs. load, etc.). The exhaust emission characteristics and concentrations can also change as conventional petroleum diesel is blended or fully substituted with renewable diesel fuels such as fatty acid methyl ester (FAME) fuels and hydrotreated vegetable oil (HVO) (Prokopowicz et al. 2015).

For larger epidemiological studies, there is an interest to use single, inexpensive, simplified exposure metrics, such as passive sampling of gas-phase NO_2_ in the breathing zone or urine sampling of selected PAH metabolites. It is thus crucial to investigate the correlation between these exposure metrics and others, such as EC and personal breathing zone sampling of multiple PAHs, for exposure assessment in epidemiological studies. Only a few studies on airborne PAH exposure in underground mines exist (McDonald et al. 2002; Seidel et al. 2002), but none used vehicles with modern engines or emission abatement technology. Individual exposure to PAHs can also be assessed via biomonitoring of urinary mono-hydroxylated metabolites. This reflects exposure from multiple routes and thus may better represent the internal dose, where mono-hydroxylated metabolites of PAHs are measured in urine (Alhamdow et al. 2017). It is also important to determine real-world emission factors (i.e., emissions per unit of fuel or energy) in the mining environment, to understand the performance in relation to the emission standards, as has been done in many studies in ambient air (Grieshop et al. 2006; Larson et al. 2017; Quiros et al. 2018).

The overall aim of this exploratory study was to qualitatively assess how the vehicle emission standard legislation and fuel affected the personal exposures and ambient concentrations in a modern underground mine. This was achieved by (1) characterizing and comparing the ambient concentrations and personal exposures to diesel exhaust for multiple exposure metrics (NO_2_, EC, eBC, PAHs), and (2) determining the correlation between said exposure metrics in a modern underground mining environment. In addition, the underground ambient concentrations were converted to real-world emission factors and related to the emission reduction technology. Moreover, we investigated the total PN and size distribution (down to 5 nm) in the underground ambient air. The miners’ personal exposure was also assessed by measuring urinary metabolites of PAHs as biomarkers of exposure. Furthermore, biomarkers of diesel toxicity were evaluated in post-shift urine: 4-HNE-MA reflecting lipid peroxidation (Dalleau et al. 2013), 8-oxodG reflecting DNA oxidative damage (Møller and Loft 2010), and 3-HPMA a marker for acrolein (Higashi et al. 2016).

## Methods

### Workplace

The study was carried out in an underground iron ore mine in northern Sweden. The main transportation route in the mine is via the approximately 600 km-long underground road network mainly using diesel-driven vehicles. The main level is located at a depth of 1365 m, and measurements (personal and ambient) were performed at levels between 907 and 1108 m below ground. The miners included in the study worked underground with either production (preparation, loading and transport) or tunnel infrastructure maintenance (ventilation, electrical, roads, maintenance). Three sampling campaigns were performed (participants not fully overlapping, details in Table S1); (1) detailed airborne exposure (*n* = 12) and ambient measurements in their near vicinity, (2) biological sampling (*n* = 27), and (3) self-administered repeated NO_2_ sampling (after the main measurement campaign) (*n* = 19 in total). In addition, ambient measurements were performed during 1 day in an emission test facility (located above ground) where underground heavy-duty vehicles such as trucks and light commercial vehicles were regularly tested. No workers used personal breathing protection, but all workers underground carried NO_2_ and CO gas alarms.

### Vehicles and fuels

In the mine, two types of diesel-powered vehicles were used to load and transport iron ore to the shafts in the areas studied: underground loaders (Stage IIIB, Stage IV, Stage V, 300 kW) and trucks (Euro VI, similar to U.S. 2010 standards). The European non-road engine emission standards Stage IIIB and IV are largely harmonized with U.S. Tier 3 and 4, while no US equivalent to Stage V currently exists. According to the mining company, all heavy equipment vehicle cabins were equipped with particle and gas filters which were replaced after 250 operation hours. The cabin particle filters in the vehicles were stated to have a filtration efficiency of 97% for incoming particles > 0.1 µm, and the gas filters were stated to reduce the incoming gases (e.g., CO, NO_x_, ammonia, radon) by 75%. All vehicles used for transportation of employees in the mine were diesel powered. The vehicles were normally fueled by Swedish low-sulfur diesel (MK1), but during one measurement day, a blend of 30% renewable diesel (HVO30) was used as fuel in one of the underground loaders (Stage IIIB). There was a mechanical ventilation system in the mine with both supply and exhaust air.

### Overview of the mining process

In the mine, the iron ore (magnetite) was extracted by means of sub-level caving. Drilling and blasting were used to extract the ore in the drifts (the production tunnels/areas following the vein of ore). In drifts 1, 3, 4 and 5 the iron ore was removed by underground loaders (Stage IIIB and IV) and dumped into vertical shafts. In drift 2, iron ore was loaded onto trucks (Euro VI) by an underground loader (Stage V) and transported to shafts further away in the mine.

### Sampling strategy

The aerosol measurements and biological sampling of urine were performed during a 1-week campaign in the fall of 2019. Follow-up self-administered NO_2_ sampling on two occasions (1 month interval between) was carried out in the following four months. The study was designed to explore and characterize different exposures in relation to work tasks in the mine where different diesel vehicles were in use. The number of participants of the different sampling groups (detailed airborne exposure, urine collection, repeated NO_2_) were based on practical limitations (number of volunteers, instruments, workplace accessibility etc.).

Table 1 presents an overview of the aerosol measurements performed. Stationary measurements in the underground ambient zones (UAZ) were conducted together with exposure measurements in the personal breathing zones (PBZ). Because the stationary UAZ measurements were performed in fixed stations, the distance between the instruments and emission sources varied during the day from around 10 to 100 m. Full-shift exposure measurements (average 7.5 h) of NO_2_, EC, eBC, and PAHs were performed in the breathing zone of 12 miners to characterize their occupational exposure to diesel exhaust. For a sub-group of three miners, repeated breathing zone exposure measurements were performed on two consecutive days with similar work tasks. The 12 volunteering miners were selected to represent different work tasks in the mine and using different classes of diesel vehicles, working the day shift. Biological sampling was performed in a group of 27 volunteering miners working shifts underground with or around diesel vehicles. Ten miners participated in both studies. In these 10 miners, biological sampling was performed on the same day as the personal air sampling. Repeated self-administered NO_2_ sampling was performed on two occasions, by 12 and 11 miners, respectively. The group of miners performing the self-administered NO_2_ sampling consisted of both the same individuals who participated in the airborne sampling as well as additional volunteering miners working shift underground (Table S1).

**Table 1:** Sampling strategy for the detailed airborne personal exposure (*n* = 12) and underground ambient measurements. Exposure and aerosol metrics were: equivalent black carbon (eBC), elemental carbon (EC), nitrogen dioxide (NO_2_, expert sampling), and polycyclic aromatic hydrocarbons (PAHs). Particle number (PN) concentration in the range 5–1000 nm, particle number size distribution (PSD), nitric oxide (NO) and carbon dioxide (CO_2_) were also measured in the ambient zone. Additional repeated NO_2_ samplings (self-administered) were performed after the main campaign

Measurement zone	Direct-reading instruments	Filter-based methods	Passive methods
Personal breathing zone (PBZ)	eBC	EC_RESP_	NO_2_, PAH_PASSIVE_
Underground ambient zone (UAZ)	eBC, PN, PSD, NO, NO_2_, CO_2_	EC_RESP_, PAH_ACTIVE_ (for calibration), total dust	NO_2_, PAH_PASSIVE_

*EC*_*RESP*_ respirable fraction of EC, *PAH*_*ACTIVE*_ active sampling of PAH for calibration of PAH_PASSIVE_ samples, *PAH*_*PASSIVE*_ passive air sampling of PAH with PUF_CYL_

### Exposure groups

For the detailed airborne measurements (Table 1), the miners were divided into three groups depending on their specific work task and type of vehicle they were operating: (1) “Tunnel Service”, (2) production loading using Stage IIIB–IV loader (”Stage IIIB–IV”), and (3) production loading using Stage V loader and Euro VI trucks (“Stage V and Euro VI”). At the time of the study, the workers in group 1 represented half of the miners working with similar tasks that shift (out of two shifts a day), workers in group 2 represented all the miners working with diesel loaders at one shift (out of two shifts a day), and workers in group 3 represented around ¼ of workers with similar work tasks (but used a more modern loader than the other teams). The workers’ detailed activity was not logged. The “tunnel service” group worked with various tunnel infrastructure maintenance service tasks in the tunnel system. They spent approximately 50% of their working time outside their vehicles. The other miners worked directly with production loading in the drifts using loaders and heavy-duty trucks (Emission standards: Stage IIIB and IV, Stage V and Euro VI). They spent majority of their working time inside the vehicles. For the repeated self-administered NO_2_ sampling, the workers were either working with “Tunnel Service” or “Drift operation”, and no detailed info on vehicle emission standards was enclosed for this larger group. Drifting (construction of drifts and transportation tunnels) and production loading were the work tasks included in the “Drift operation” group. The workers may have had slightly different work tasks (within the main work division) on the repeated NO_2_ sampling, but no detailed work log was kept.

The urine samples were collected from miners working shift underground, with various tasks including tunnel service, production and drift operation but no detailed work description or vehicle emission standards was collected.

### Direct-Reading instruments

Portable aethalometers (microAeth model AE51 and model MA200, AethLabs, CA, USA) with a pre-separator (microCyclone, AethLabs) with an aerodynamic particle size cut point of 1.6 µm were used to measure eBC mass concentrations in the PBZ. The time resolution of the aethalometers was 30 s and the flow rate was set to 100 mL/min. With the AE51, the filter strip was replaced at least twice during each work shift in order to minimize the filter loading artifact (Good et al. 2017). In addition, a post-processing filter loading correction (based on the measured attenuation) was performed on eBC data from the AE51 (Kirchstetter and Novakov 2007; Good et al. 2017). On average, the correction increased the eBC values by 27 ± 16%. No filter loading correction was applied to the data from MA200 as it uses internal real-time filter loading correction (Drinovec et al. 2015).

A multiwavelength aethalometer (model AE33, Magee Scientific, Slovenia) with a particle size cut off point of 3.5 µm (≈ respirable fraction) was used in the UAZ. The time resolution was 1 s and a wavelength of 880 nm was used to estimate eBC concentrations, using the standard settings of particle optical properties recommended by the manufacturer. Calibration before the campaign with diluted fresh diesel exhaust from a Stage IIIB loader (23 kW) yielded an eBC_AE51_/eBC_AE33_ ratio of 0.74 ± 0.06. The eBC/elemental carbon (EC) ratios were 2.8 for eBC_AE33_/EC, 2.2 ± 0.2 for eBC_AE51_/EC. No comparison of AE33 and MA200 were possible; however, a recent instrument comparison showed a 9% lower eBC yield for MA200 compared to AE33 (Blanco-Donado et al. 2021).

PN and size distribution in UAZ (5–1000 nm) was measured with a fast mobility particle analyzer (model DMS500, Cambustion Ltd., UK) using the inversion matrix for diesel soot agglomerates. NO and NO_2_ in UAZ were measured with a chemiluminescence analyzer (CLD 700 AL, ECO PHYSICS AG, Switzerland) and the CO_2_ concentration in UAZ was measured with a non-dispersive infrared CO_2_ analyzer (LI-8020, LI-COR, MI, USA).

### Filter-based methods

#### Sampling and analysis of EC

Time-integrated respirable fractions of EC were collected in PBZ and UAZ according to NIOSH NMAM 5040 (NIOSH 2003). Samples were collected on 37 mm quartz filters (SKC Inc., PA, USA) mounted in conductive three-piece filter cassettes (SureSeal, SKC Inc.) with cyclones (BGI4L, BGI Inc., USA; cutoff 4 µm). Pumps (Apex2, Casella, UK) were used to provide the sample flow rates set at 2.2 mL/min. A primary calibrator (TSI Model 4100 Series. TSI Inc., MN, USA) was used to regularly control the air flow rates. The filters were stored in a refrigerator (+ 6 °C) until analysis and analyzed for EC with a thermal optical analyzer (DRI Model 2001 OC/EC Carbon Analyzer, Atmoslytic Inc., CA, USA) using the NIOSH NMAM 5040 diesel exhaust protocol (2003). The limit of detection (LOD) for EC was 0.06 µg C cm^−2^.

#### Sampling and analysis of total dust

Total dust mass was sampled with a flow rate of 2 L min^−1^ on a 37-mm Teflon filter (Teflo, pore size 2 µm, Pall Corporation, NY, USA) mounted open face in conductive three-piece filter cassettes (SureSeal, SKC Inc., PA, USA). The filter samples were analyzed gravimetrically for determination of total dust (LOD was 0.05 mg/sample).

### Passive methods

#### *Sampling of NO*_*2*_

Passive sampling of NO_2_ was performed in the PBZ and UAZ. The sampler was mounted onto a holder and placed in the PBZ of the worker. The measurement method is based on diffusion of NO_2_ (Ferm and Rodhe 1997; Ferm and Svanberg 1998), and the accredited analysis of the NO_2_ samples was performed by IVL Swedish Environmental Research Institute. Method LOD was 10 µg m^−3^ using 8 h sampling and measurement uncertainty was 10%. Samplers were stored in sealed plastic containers before and after sampling. Temperatures during the measurements in the mine ranged from 20.4 to 24.1 °C. Self-administered passive samplings of NO_2_ were performed by 11–12 miners themselves during a full work shift on two occasions after the main measurement campaign, with at least a month interval between. The workers received written instructions of how to perform the sampling and filled in details of sampling times, temperature, work tasks, and work depth (level).

#### Sampling of airborne PAHs

A detailed description of sampling and analytical methodologies of airborne PAHs can be found in the Supplemental Information. In brief, the airborne PAH concentrations of the 16 US EPA PAHs (Hussar et al. 2013) were determined in the PBZ and UAZ with a passive collection method. This passive method is based on cylindrical polyurethane foam samplers (PUF-cyl, length: 10 cm, diameter: 2.2 cm) (Strandberg et al. 2018).

The PUF-cyl passive sampler has previously been evaluated and calibrated with uptake factors for short sampling times (2–8 h) in other work environments (Bohlin et al. 2010; Strandberg et al. 2018). Concurrent sampling with the passive PUF-cyl sampler and active sampling (NMAM 5515, (NIOSH 1994)) in the UAZ were performed to determine the uptake-rates of PUF-cyl in the present work environment. The uptake factors were then used to determine the PAH levels from the passive sampling in the PBZ.

The PUF-cyl samplers were extracted using an Accelerated Solvent Extractor (Dionex ASE 350; Thermo Fisher Scientific), purified using an open column (ID 0.9 cm) with 1 g sodium sulfate, 4 g activated silica and 2 g activated alumina. PAHs were separated and detected by means of high-resolution gas chromatography/low resolution mass spectrometry (HRGC/LRMS) (Agilent Technologies, Inc.,CA, USA).

#### Emission factors

Real-world emission factors (in units g pollutant per kg burned fuel) were estimated by assuming that all pollutants originate from the vehicles. The emission factors were calculated by Eq. (1) by assuming complete combustion (Kirchstetter et al. 1999; Gordon et al. 2014) and that all carbon in the fuel was combusted to CO_2_:

1 $$\mathrm{Emission\, factor }(\mathrm{EF})={10}^{3}\frac{\left[\mathrm{Pollutant}\right]}{\Delta \left[{\mathrm{CO}}_{2}\right]\frac{{\mathrm{MW}}_{\mathrm{C}}}{{\mathrm{MW}}_{{\mathrm{CO}}_{2}}}} {C}_{\mathrm{f},}$$

where the concentration of [pollutant] and ∆[CO_2_] are in µg m^−3^. The ∆[CO_2_] is the background corrected CO_2_ concentration. The background concentration was measured before the start of the work shift when no activity was performed in the stationary underground ambient measurement zone. The carbon mass fraction (*C*_f_) of diesel 0.85 was used for all days (Gren et al. 2021).

### Biological monitoring of miners

The study was approved by the Regional Ethical Committee of Lund University, Sweden (registration no. 2009–0568) and performed in accordance with the Declaration of Helsinki, including obtaining informed written consent from all subjects. Miners between 20 and 60 years of age who had not smoked during the last six months were invited to participate in the biological sampling. Spot urine was collected from all 27 miners after their 8 h work shift, and 10 of these miners were also monitored for air exposure. Urine samples were transported at 4 °C to the research laboratory, where urine density was measured, whereafter the urine samples were stored at − 20 °C prior to analysis.

### Analysis of urinary biomarkers

Urine samples were analyzed for PAH metabolites: 2-naphthol (2-Nap); 2,3-hydroxyfluorene (2,3-OH-Flu); 2,3-hydroxyphenanthrene (2,3-OH-Phe); 1-hydroxyphenanthrene (1-OH-Phe); 4-hydroxyphenanthrene (4-OH-Phe); and 1-hydroxypyrene (1-OH-Pyr). Additionally, we analyzed for 3-hydroxypropyl mercapturic acid (3-HPMA), a marker for acrolein; 4-hydroxynonenal mercapturic acid (4-HNE-MA), a marker for lipid peroxidation; and 8-oxo-2′-deoxyguanosine (8-oxodG), a marker for DNA damage. Method details are described in Krais et al. (2021) and in Supplemental Information.

The samples were analyzed using liquid chromatography–triple quadrupole linear ion trap mass spectrometry (LC–MS/MS; QTRAP5500, AB Sciex, MA, USA). 2-OH-Phe and 3-OH-Phe, as well as 2-OH-Flu and 3-OH-Flu, could not be separated and were therefore analyzed as single peaks (∑2,3-OH-Phe and ∑2,3-OH-Flu, respectively). All biomarker concentrations were adjusted to urinary density (ng/mL urine).

### Statistics

The statistical analysis was performed using IBM SPSS Statistics 26 software. The personal exposure data was lognormally distributed (assessed with Q-Q plots). For correlation tests of exposure metrics, linear regression was performed on log-transformed data. Pearson correlation was used for tests with only ambient zone measurements (*n* = 4–5) as this data was normally distributed (assessed with Q-Q plots and Shapiro–Wilks normality test). Wilcoxon signed rank test were used to compare expert and self-administered passive NO_2_ measurements. An average NO_2_ exposure for each miner was then used to calculate the group average. For all tests, values below LOD were set to half the LOD and *p* values < 0.05 were accepted as significant.

## Results

### Exposure levels and correlations between exposure metrics

#### Personal exposures and underground ambient aerosol measurements

**Fig. 1:**
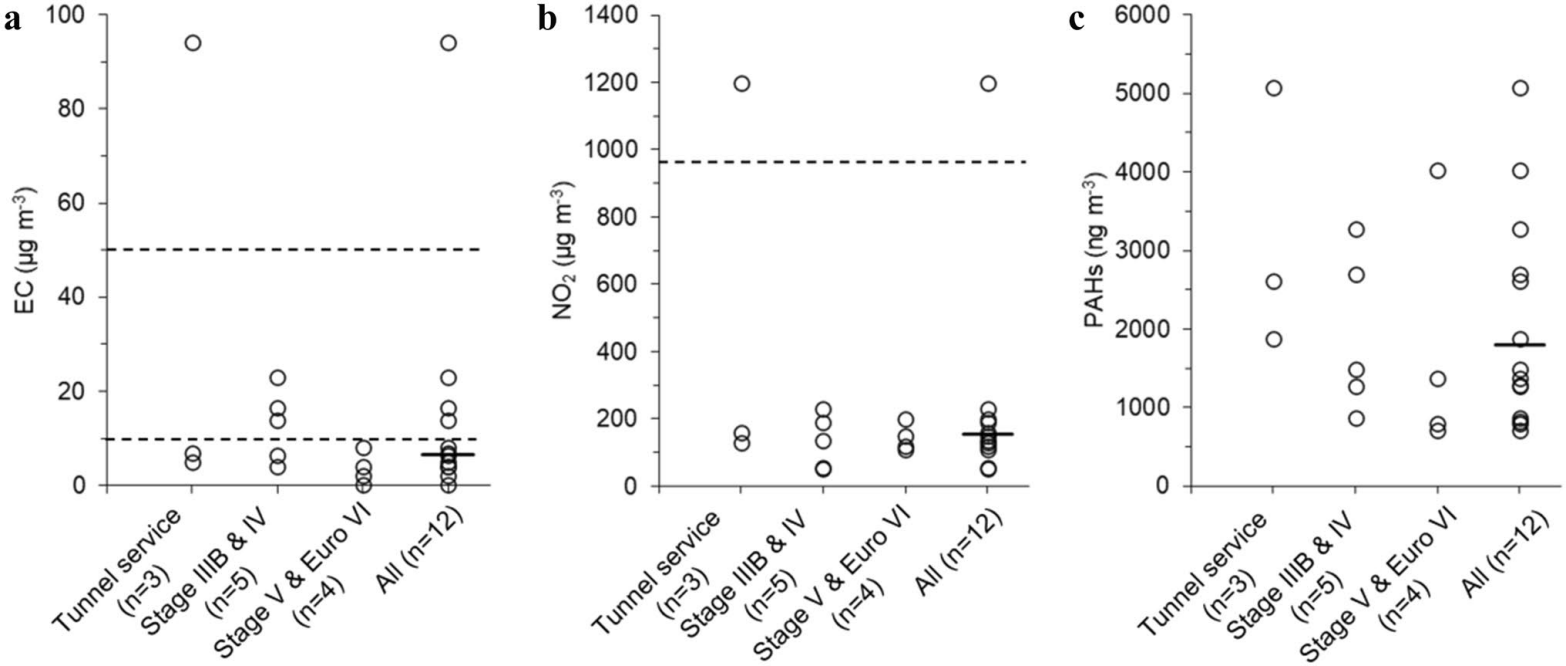
Summary of the personal exposure concentrations of **a** EC, **b** NO_2_, and **c** PAHs (sum of 16 US EPA PAHs). The black lines show the geometric mean of each group. The miners were divided into three groups depending on their work task and type of vehicle. The “Tunnel service” group works mostly with maintenance in the transportation tunnels (not in the drifts). “Stage IIIB and IV” and “Stage V and Euro VI” groups work with production loading in different drifts; the names of these groups refer to the EU emission standard class of their vehicles (loaders or trucks). Stage V (loaders) and Euro VI (trucks) levels are more stringent than Stage IIIB and IV (loaders) levels. “All” summarizes the three groups. Dashed lines show the underground OELs: for EC, 50 µg m^−3^ (in EU from 2026), and 10 µg m^−3^ (EC OEL in Denmark and Netherlands). For NO_2_, 960 µg m^−3^ (in EU from 2023). No repeated measurements are included in this figure, and for the three miners in which we measured on two consecutive days (Table S2) the average values are used

**Fig. 2:**
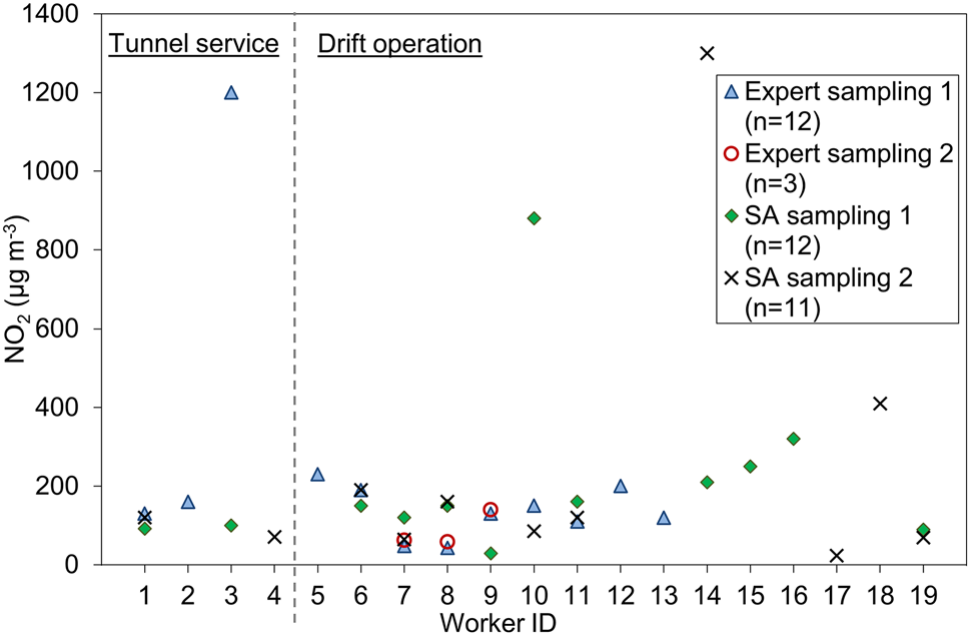
Measurements of NO_2_, including repated measurements, were performed by either occupational hygienists (Expert sampling) or as self-administered sampling (SA sampling). Miners with workers ID no. 1–4 worked with tunnel service and 5–19 worked in the drifts with production preparation, loading and transport

The group-level average exposures to elemental carbon (EC), NO_2_, and PAHs (sum of 16 US EPA PAHs) are shown in Fig. 1. The participating underground miners had an average [geometric mean (GM)] EC exposure concentration of 7 µg m^−3^ [geometric standard deviation (GSD): 2.7]. One miner (worker ID no. 3) who worked with maintenance in the tunnels was exposed to an average concentration of 94 µg m^−3^ of EC as well as to a high concentration of NO_2_ (1200 µg m^−3^, Table S2). On average, the underground miners were exposed to NO_2_ levels of 153 µg m^−3^ (GSD: 2.1). Repeated self-administered NO_2_ measurements were performed by 19 miners (Fig. 2), and the average exposure (GM) was 165 µg m^−3^. No differences were found between the expert- and self-administered measurements (*p* > 0.3).

In the group of miners operating underground loaders with the older emission standard (Stage IIIB and IV), three out of five were exposed to EC concentrations above 10 µg m^−3^, while none the miners operating vehicles of newer emission standard (Stage V loader and Euro VI heavy-duty trucks, Fig. 1a, b) were exposed to levels above 10 µg EC m^−3^. The individual exposure, measured in the personal breathing zone (PBZ), and the aerosol concentrations in the stationary underground ambient zone (UAZ) are summarized in Table S2.

The average PAH exposure was 1790 ng m^−3^ (Fig. 1c, Table S2). On average, naphthalene contributed to around 60% of the total PAH exposures measured with the passive sampler (Table S3). To compare the urinary metabolite analysis with the corresponding parent PAH in air, the individual exposure concentrations of naphthalene, fluorene, phenanthrene, and pyrene are shown in Table S4.

Repeated measurements in the same individuals for similar work tasks (workers ID no. 7, 8, 9) showed good agreement (less than ± 20% deviation of means of EC and NO_2_) of exposure levels between days (except for PAHs where the deviation of means was 45% or higher).

### Correlations between exposure metrics

**Fig. 3:**
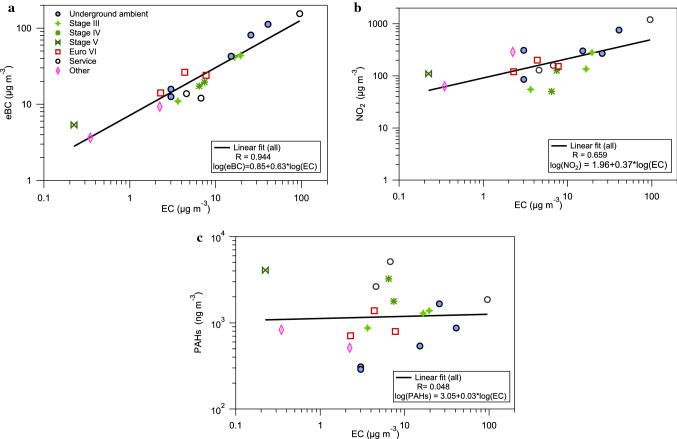
Correlations in underground ambient zones and PBZ between **a** eBC, **b** NO_2_, and **c** PAH concentrations and EC concentrations. The measurements in PBZ are divided into several subgroups denoting the emission standards of the mining vehicle they operated in the drift (Stage III, IV, V or Euro VI). The “Service” group worked with tunnel maintenance. PAHs are the sum of the 16 US EPA PAHs. The data were log transformed and the lines show the linear regression model. The correlation was statistically significant for eBC (*R* = 0.94; *p* < 0.001) and for NO_2_ (*R* = 0.66; *p* = 0.002). No statistically significant correlations were found between PAHs and EC (*p* > 0.3). NO_2_ and PAHs were measured with passive sampling techniques, EC was measured with an offline filter-based method, and eBC with highly time-resolved instrumentation (micro-aethalometer). No repeated measurements are included in this figure, and for the three miners in which we measured on two consecutive days (Table S2) the average values are used

Strong positive correlations (*p* < 0.01) of measured air concentrations were found between eBC and EC, as well as between NO_2_ and EC, while no correlation was seen between PAHs and EC (Fig. 3). The time-integrated filter-based measurement technique of EC was compared to the shift average of the direct-reading measurement technique of eBC (Fig. 3a) and showed strong correlation (*R* = 0.94, *p* < 0.001). However, the eBC concentration was consistently higher than EC, and the average ratio (GM) of eBC/EC was 3.5. By excluding the low EC exposed miners (workers ID no. 6 (day 2), 11) the eBC/EC ratio was slightly lower (GM:3.0). The correlation coefficient between NO_2_ and EC was *R* = 0.66 (*p* = 0.002), and the average (GM) NO_2_/EC ratio was 22 (Table S2).

**Fig. 4:**
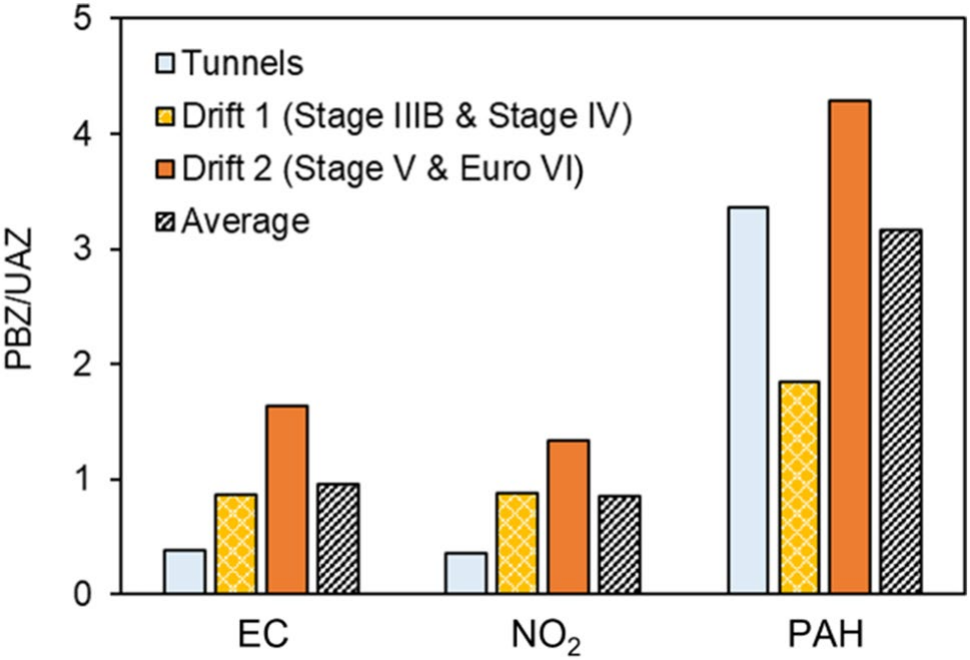
The measurements in the personal breathing zones (PBZ) compared to the measurements in the underground ambient zones (UAZ) for three locations in the mine. For the PBZ measurements the geometric means of the workers in each location are used. In Tunnels *n* = 3, in Drift 1 *n* = 3, in Drift 2 *n* = 4. PAHs are the sum of the 16 US EPA PAHs

The ratios between the measurements in the PBZ and the UAZ for EC, NO_2_, and PAHs for the miners as the full group (average) and at the three underground locations (tunnels, drift 1, and drift 2) are shown in Fig. 4. The average PBZ/UAZ ratios were strikingly similar for NO_2_ measured with the passive sampler and EC (within + − 20%) measured with the active sampler and relatively close to 1. This shows that the precision in these measurements was very high. For PAHs, a completely different pattern was found, with significantly higher PBZ/UAZ ratios (≈ 3). In addition, no correlations between the PAH and EC measurements were found (Fig. 3c) when all measurements were included, but a trend of PAHs increasing with EC was found when only looking at the UAZ data (*R* = 0.87, *p* = 0.052).

### Real-time equivalent black carbon (eBC) exposure

**Fig. 5:**
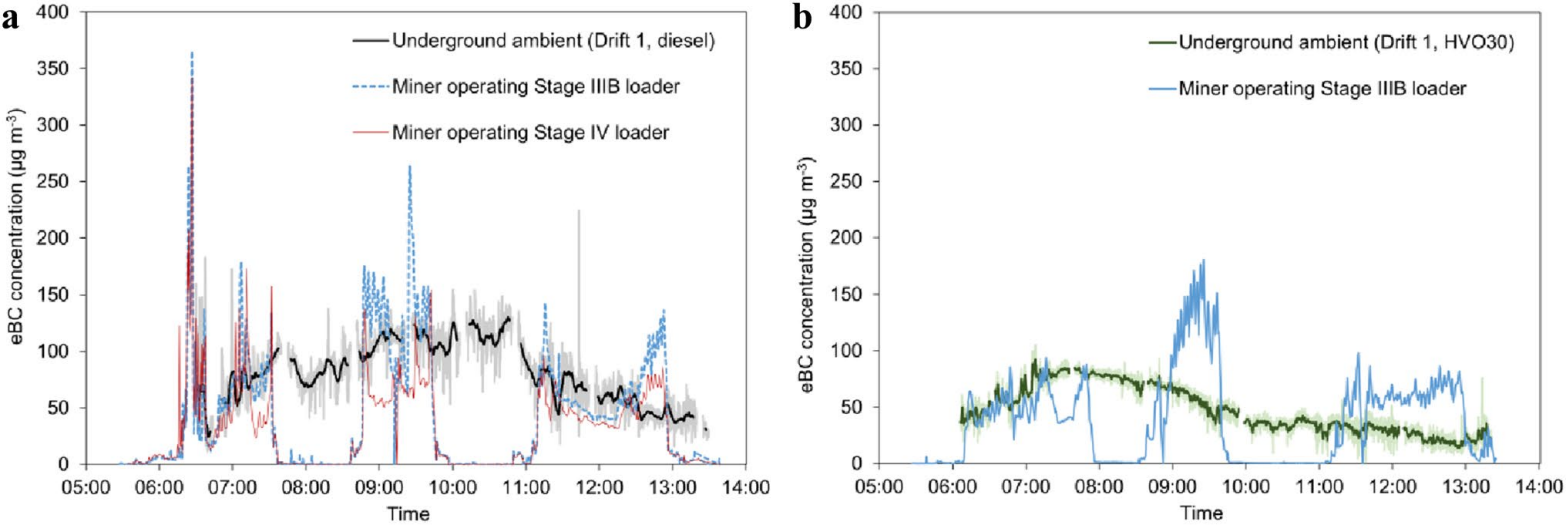
eBC concentrations in drift 1 measured in the underground ambient zone and PBZ of the miners. In **a** the two underground loaders in the drift operated on petroleum diesel. In **b** the single underground loader operated on HVO30 (only one loader this day). Only PBZ for miner operating the Stage IIIB loader (worker ID no. 5) is included in (**b**) as the miner operating the Stage IV loader (worker ID no. 6) did not work in the drift during the HVO30 test due to vehicle malfunction. The miners were moving the ore from the drift to the shafts, which were located within the drift. The concentration in the ambient zone in the same drift is given with 1 s resolution (shaded areas) and 1 min moving average. The PBZ exposure is measured with 30 s resolution

Highly time-resolved personal exposure to eBC in drift 1, and the concentration in the underground ambient zone, are shown for two separate days in Fig. 5. On the first day, two miners worked full-time transporting iron ore using Stage IIIB and Stage IV loaders running on diesel (Fig. 5a). On the second day, only the Stage IIIB loader was in operation, running on HVO30 (Fig. 5b). The miners in drift 1 had work breaks around 7:30 and 10:00 both days, when they left the drift and sat inside the underground break room. This is observed in Fig. 5 as sharp decreases in eBC concentrations in the PBZ. During their time inside the vehicle cabins on the first day, both miners were exposed to high levels of eBC. On average they were exposed to 64 and 46 µg m^−3^ (compared to 72 µg m^−3^ in UAZ) for the miner in the Stage IIIB and IV loader (both running on diesel), respectively. These miners had short peaks of higher eBC exposure when both underground loaders ran on diesel (Fig. 5a), compared to when only the one Stage IIIB underground loader ran on HVO30 (Fig. 5b).

The eBC concentration was on average substantially lower in drift 2 where vehicles of more stringent emission classes were used compared to drift 1 (Table S2, Fig. S1). However, there was no clear decrease in eBC seen during the work breaks (from around 09:45), which could be due to poorer filtration efficiency in the supply air to the underground break room used by the workers in drift 2. The miner operating the Stage V loader in drift 2 had on average very low eBC exposure (5 µg m^−3^), but with short peaks of higher concentration (> 50 µg m^−3^). The miners driving the Euro VI trucks in drift 2 were on average exposed to eBC concentrations below 50 µg m^−3^, but with multiple peaks at higher concentrations (Fig. S1).

### Particle size distributions and real-world emission factors

**Fig. 6:**
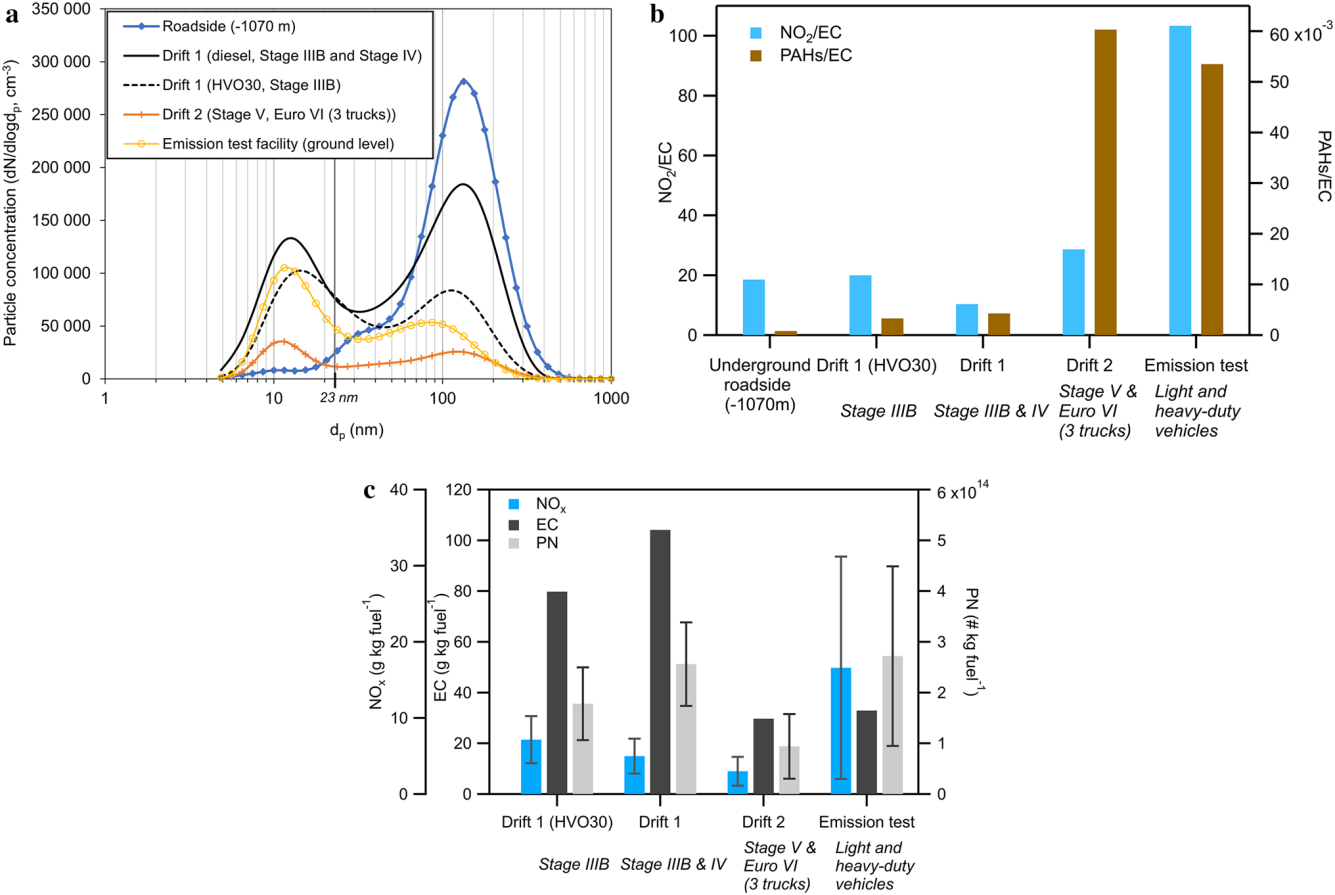
**a** The average particle number size distribution measured in the stationary ambient zones concurrent with the PBZ measurements (around 7.5 h) at the roadside, the two drifts, and at the emission test facility (ground level). The measurements in the emission test facility were measured over 3 h. In **b**, the ratios NO_2_/EC and PAHs/EC are shown for all ambient zones. PAHs are the sum of the 16 US EPA PAHs. **c** Average real-world emission factors (per kg fuel) of NO_x_, EC, and PN (measured as the soot mode concentration, which approximates the solid fraction of PN > 23 nm) in the drifts and at the emission test facility. The error bars in (**c**) represent ± 1 std. dev. of the variation throughout the measurement day (not available for EC)

The average PN size distributions (0.005–1 μm) measured at the stationary ambient zones during the work shifts are shown in Fig. 6a. In the drifts and at the emission test facility, the size distribution was composed of two modes: nucleation (around 15 nm) and soot (around 100 nm). At the roadside, the larger soot mode particles dominated the number concentration (nucleation mode < 10% of total concentration). In the drifts, the nucleation mode particles (not included in the EU emission standard of PN) instead contributed to around 40–55% of the total number concentration (0.005–1 μm). In drift 2, the average number concentration was low throughout the day for both the nucleation and accumulation mode. In drift 1, there were significant peaks of nucleation mode particles (geometric mean diameter [GMD] 12–16 nm) during the first hour of the work shift when the underground loaders were started. Similarly, a high ratio of nucleation mode to accumulation mode particles was found at the emission test facility where vehicles were operated in idle mode. The emission test facility also had the highest ratios of PAH/EC and NO_2_/EC (Fig. 6b). PAH/EC ratios were highest for drift 2 where the most modern vehicle types were used.

The drift 1 measurements were performed over two days. The underground loaders (Stage IIIB and IV) operated on diesel the first day, and HVO30 the second. However, due to a vehicle malfunction, only the Stage IIIB loader was in operation on the second day, and comparisons of absolute concentrations from diesel and HVO30 operation are therefore not possible. The NO_2_/EC ratio was a factor 2 higher during the HVO30 test (Fig. 6b). Moreover, the nucleation mode particles dominated the total PN concentration (50% compared to 40% for diesel). It should be noted that there were some additional activities in both the drifts, such as road maintenance with road graders (operated with diesel) which occurred on multiple occasions during both days.

The real-world emission factors (per kg fuel) in the drifts are shown in Fig. 6c. PN was measured  as the soot mode total number concentration because this better represents the solid (EC-dominated) particles than the total particle concentration (Fig. S2). In the drifts, the lowest emission factors of PN and NO_x_ were found in drift 2 which used the vehicles with the latest emission standards for both non-road vehicles (Stage V) and heavy-duty trucks (Euro VI). In the emission test facility, where the vehicles were tested in idle mode, the highest emission factor of NO_x_ and a relatively high PN emission factor (with large variations throughout the measurement) were found.

### PAH metabolites and effect biomarkers

The summary of biomarkers measured in urine are shown in Table 2. The participants consisted of 27 miners (8 f/19 m, age 20–54 years). All were currently non-smokers (6 previous smokers). The most abundant airborne PAHs in the mine were naphthalene, acenaphthene, fluorene and phenanthrene (Table S3). Urine was analyzed for metabolites of naphthalene (2-Nap,) fluorene (2,3-OH-Flu), phenanthrene (2,3-OH-Phe, 1-OH-Phe, 4-OH-Phe), and pyrene (1-OH-Pyr). The PAH metabolites were detected in all but three subjects (Table S4). Of the 10 miners on whom we performed both airborne exposure measurements and biological monitoring, no significant correlations between individual PAHs in the air and the respective urinary metabolite were found (*p* > 0.07, *N* = 10), neither did we find any correlations between EC, eBC, NO_2_ or the 16 US EPA PAHs and PAH metabolites concentration (*p* > 0.09, *N* = 10).

**Table 2:** Summary of the density-adjusted urinary biomarkers from 27 occupationally exposed miners

	Avg. ± Std. Dev	Median	Maximum	Percentiles	
				25th	75th
PAH metabolites (ng mL^−1^)^c^					
2-Nap	4.20 ± 2.85	3.89	8.90	1.80	7.35
ΣOH-Flu^a^	0.53 ± 0.5	0.36	2.52	0.25	0.70
ΣOH-Phe^b^	1.64 ± 1.16	1.29	6.97	1.09	1.66
1-OH-Pyr	0.51 ± 0.72	0.31	4.02	0.27	0.48
Other biomarkers (ng mL^−1^)^c^					
4-HNE-MA	89 ± 63	83	226	40	135
8-oxodG	8.1 ± 3.5	7.3	18.3	5.6	9.1
3-HPMA	1000 ± 820	790	3170	450	1160

Individual urinary concentrations are presented in Table S4

^a^Sum of 2- and 3-OH fluorene

^b^Sum of Σ2,3-OH phenanthrene, 1-OH phenanthrene and 4-OH phenanthrene

^c^The urinary metabolites were adjusted for urine density

Additionally, we analyzed 3-hydroxypropyl mercapturic acid (3-HPMA), a marker for acrolein (exposure and endogenously formed); 4-hydroxynonenal mercapturic acid (4-HNE-MA), a marker for lipid peroxidation; and 8-oxo-2′-deoxyguanosine (8-oxodG), a marker for DNA damage. All these biomarkers were detected in all subjects (Table S4). Of the ten miners on whom we performed both airborne exposure measurements and biological monitoring, urinary 3-HPMA was found to correlate with airborne NO_2_ (*R*_S_ = 0.68, *p* = 0.03), benzo[a]pyrene (*R*_S_ = 0.74, *p* = 0.014), and phenanthrene (*R*_S_ = 0.72, *p* = 0.02) (Fig. S3a–c). In addition, urinary 3-HPMA correlated with urinary 8-oxodG (*R*_s_ = 0.43, *p* = 0.03, *N* = 27) (Fig. S3d).

## Discussion

### Exposure and aerosol characteristics

#### Personal exposures

The participating miners had an occupational exposure to NO_2_, EC and PAHs (Table S2), and the exposures were on average below future EU OELs (Fig. 1). However, all workers except two were exposed to EC levels well above 1 µg m^−3^ (GM: 7 µg m^−3^), which poses a substantial excess lifetime risk of lung cancer (Vermeulen et al. 2014; Ge et al. 2020) for exposures over a full working life. In addition, a few workers were above, or unsatisfactorily close to the future European OELs of EC and NO_2_, which suggests there might be a risk of exposure above the OELs. Compared to the newly implemented more stringent OELs of EC in Denmark and Netherlands (10 µg m^−3^), 5 out of 12 miners were exposed to higher concentrations, and all but one exceeding one-third of these more stringent OELs (Table S2). In an earlier study in a Swedish iron ore mine where the test subjects performed similar underground work tasks as in this study, the average EC (assessed using “colorimetric analysis”) and NO_2_ exposures were 27 µg m^−3^ and 280 µg m^−3^, respectively (Ädelroth et al. 2006). Compared to our results the mean EC exposure of the participating miners has decreased by 74% (GM: 7 µg m^−3^), and the NO_2_ exposure by 46% (GM: 153 µg m^−3^). Including the larger group of miners performing the self-administered NO_2_ sampling, the average was slightly higher (165 µg m^−3^). One miner working with tunnel service (ID no. 3) was exposed to levels exceeding the measured EC and NO_2_ range in the previous study. Even if the EC exposure in this mine was on average lower than recent studies in underground mines [GM: 67–110 µg EC m^−3^, (Debia et al. 2017; da Silveira Fleck et al. 2018)], reductions are necessary to limit the health risks.

#### Validation of the eBC method toward EC

The variation in eBC which was used as a proxy for EC measured with high time resolution throughout a work shift showed that many of the workers were exposed to very high peak concentrations, or concentrations higher than 50 µg m^−3^ during shorter periods (≈ 1 to 60 min), even if the average exposures were significantly lower. The correlation of the direct-reading eBC technique compared to the offline EC measurement was strong (*R* = 0.94), as was also previously found in another occupational study (waste workers *R* = 0.822, (Lee et al. 2015)). This indicates that the direct-reading instrument is useful for identifying work tasks or areas with elevated risk of EC exposure.

However, only measuring eBC might overestimate the EC concentration, as this was on average a factor 3.0 higher than EC (excluding miners with < 0.5 µg EC m^−3^), which is in line with other studies (waste worker: 1.99 (Lee et al. 2015), semi-urban areas: 2.7–3.3 (Jeong et al. 2004), HVO and diesel soot: 2.8 ± 0.4 (Gren et al. 2021). This means that the eBC measurement in this study cannot be generalized directly as an estimate of EC concentration, but can instead represent a direct-reading measurement of the EC concentration divided by a factor of three. Calibration before the campaign with diesel exhaust from a small Stage IIIB loader yielded a slightly lower eBC/EC factor of 2.2 ± 0.3 compared to in the mine.

The discrepancy between the two measurement methods are most likely due to the particles’ varying light absorption and scattering efficiency, which is dependent on chemical composition (organic carbon, brown carbon) and structure (size, intermixing state, degree of graphitization) (Kirchstetter et al. 2004; Watson et al. 2005). The discrepancy in our study between the lab calibration (using a diesel loader, 23 kW, Stage IIIA) pre-study and the occupational field measurements indicates that there may be an additional source in the mining air that disturbs either the EC or eBC signal. The most likely explanation is that iron ore rock dust from the mining process (below the 1.6 µm cut point of the AE51 and MA200) contributes to the light attenuation measured by the aethalometers by either absorption or scattering, thus presenting a positive bias in the eBC measurement. This is also consistent with the higher eBC/EC ratio for the vehicles complying with the most advanced emission legislation (Table S2).

In summary, we validated the eBC method for near real-time PBZ measurements of EC exposures in the harsh mining environment with high co-exposures of larger wear particles from the mining process and potentially high vibration load on the sensors. The correlation between online eBC measurements and off-line full-shift averages of EC exposures in the PBZ was high. However, there was an offset between the methods, which may be site or instrument model specific. Thus, in future applications of the eBC method we always recommend collecting an EC filter sample in parallel.

#### Correlations between airborne exposure metrics (EC vs NO_2_)

The correlation between NO_2_ and EC was also relatively strong (*R* = 0.66) and in line with other studies at similar workplaces (Hedmer et al. 2017; Berlinger et al. 2019) indicating that NO_2_ measured with the passive sampler can be used as a reasonable proxy for EC in, for example, epidemiological studies. However, a major difference was that the NO_2_/EC ratio (GM: 22) was five times higher than previously reported from an underground mine (Berlinger et al. 2019). The higher ratio is caused by the lower EC exposure in our study, which is hypothesized to be due to the higher level of emission abatement strategies (internal or external emission reduction) in the engines of the vehicles used in this mine. Diesel particulate filters (DPFs) in particular are known to be efficient in removing PM including EC (Bugarski et al. 2020; Zeraati-Rezaei et al. 2020; Gren et al. 2021). The operators of the relatively older machines (from 2012 and forward, Stage IIIB and IV) in the mine had a lower NO_2_/EC ratio (GM:10) than the operators of vehicles with the latest emission standard (GM:71, Stage V and Euro VI). The NO_2_ and EC concentrations, their correlation, and the ratio between the two exposure markers may continue to change as the vehicle fleet in mines is renewed and vehicles complying with later emission standards are implemented. It should be noted that the ore blasting in this mine creates by-products such as NO/NO_2_ (and CO) which can increase the NO_2_ concentration in the mine. However, blasting was only performed night-time outside working hours of the studied workers. None of the studied workers or stationary measurements were performed in the vicinity of active blasting sites or before ventilation of blasting gases was complete. The NO was < 0.01 ppm and NO_2_ was < 0.001 ppm at the start of each measurement day.

#### Personal exposure vs underground ambient measurements

The comparison of the average personal exposure and the concentrations in the underground ambient zones—PBZ/UAZ ratios—were relatively close to 1 and almost identical for EC and NO_2_ in the tunnels as well as drifts 1 and 2 (Fig. 4). The absolute values of the ratios depend to some extent on the positioning of the UAZ in relation to the emission sources and whether the workers were directly exposed to undiluted exhaust near the sources. Nevertheless, the data demonstrates that EC and NO_2_ emissions came from similar sources, and that the filtration devices of the air supply to the vehicles were of similar efficiency for both EC and NO_2_ (for workers exposed in the drifts).

For PAHs (sum of 16 US EPA PAHs), a completely different pattern was found, with higher PBZ/UAZ ratios. This illustrates that area sampling as in UAZ leads to an underestimation of personal PAH exposures. This is of concern as PAHs are carcinogenic and cardiotoxic (IARC 2014; Alhamdow et al. 2017, 2020). The high ratio in the tunnels could partly be caused by the workers having their vehicles idle during tunnel maintenance, causing higher exposures than measured at the tunnel roadside, while alternative hypotheses are needed in drift 2. The vehicles in drift 2 all followed the most stringent emission legislation of each class at the time of the study (Stage V, and Euro VI) which nominally means efficient removal of organic components, PM, PN and NOx. However, the PAH removal is not regulated and the removal efficiency might vary depending on the type of aftertreatment system (Liu et al. 2015). In addition the PAH/EC ratio was higher in drift 2 (Fig. 6b) than in the other underground areas, which suggests that the emission reduction systems were not as efficient in removing gas-phase PAHs as PM or EC. There is also a possibility that some of the non-removed semi-volatile PAHs may partitioning into the gas phase downstream some types of DPFs (Hu et al. 2013).

Another possible explanation could be due to secondary emissions of PAHs or to non-vehicular sources of PAHs. The gas-phase PAHs might have condensed and accumulated on the surfaces in the mine and possibly on clothes, indoor surfaces, and air filtrations systems in the vehicles. The built-up layer of PAHs may then at a later time-point have caused secondary emissions of semi-volatile PAHs as they are re-partitioning from solid to gas phase (Bohlin et al. 2008). This mechanism would mean that compared to NO_2_ and EC, the ventilation systems in the mine are less efficient in removing PAHs after an emission event.

#### Impact of emission reduction technology on ambient aerosol concentrations

The emission standards for Stage V (loader) and Euro VI (trucks) are more stringent than Stage IIIB and IV in terms of particles, as they have reduced PM limits and also include a PN restriction (which in practice requires the use of DPFs). This is likely the cause of the lower PN emission factor in drift 2. Similarly, Bugarski and Hummer (2020) found that the EC and PN decreased with increasing levels of aftertreatment technology in underground mining equipment. The EU vehicle PN emission standard only considers solid particles and these are measured as non-volatile particles larger than 23 nm, which in our study was approximated as the soot mode PN. It should be noted that the emission standard excludes the smaller nucleation mode (both solid and volatile) particles. These particles have a different chemical composition compared to larger solid soot particles, and are commonly dominated by lubrication oil and metal oxides (Karjalainen et al. 2016; Gren et al. 2021). This smaller mode contributed to a considerable portion of the total number concentration in this study (Fig. 6a), especially in drift 2 where the Stage V and Euro VI vehicles were in operation, but also during idle mode (emission test facility) and in the beginning of after cold start (drift 1). This indicates that these nucleation mode particles may not be as well reduced by the emission reduction units implemented to comply with improved emission legislation (Giechaskiel et al. 2014). However, the total dust mass was 1.5–3 times higher (1275 µg m^−3^) in drift 2 than in drift 1 or at the roadside (Table S2). This indicates that the particle number concentration may not only originate from the exhaust but can also be from non-exhaust emissions such as vehicle and road wear particles.

High PAH/EC and NO_2_/EC ratios were seen in the emission test facility, where light-duty vehicles and heavy-duty trucks were tested in idle mode, which facilitated lower PM emissions but higher NO_x_ and PAH emissions in comparison to EC (Fig. 6b). The vehicles tested at the emission test facility were mainly of more stringent emission standards, Euro 5–6 for the light-duty vehicles and Euro VI for the heavy-duty vehicles. The idling could have led to reduced operation temperatures in the aftertreatment system, which caused the NO_x_ reduction units (often selective catalytic reduction) and removal system for organic gases (e.g., diesel oxidation catalyst) such as PAHs to operate with lower removal efficiency (Mera et al. 2020), while the EC removal in the DPF is presumably less affected. Additionally, EC emissions are commonly low during idling. However, the emission factor of PN in the emission test facility was still relatively high due to the smaller particle size in the soot mode during idle.

#### Impact of fuel

Although no comparisons of absolute concentrations of the fuel substitution (HVO30) were possible due to vehicle malfunctioning during the tests, some observations can be made depending on either fuel- or vehicle-specific emission reduction techniques. Emission factors for PN were similar in drift 1 both days, but higher for NO_x_ during the day with only the Stage IIIB vehicle (using HVO30) in operation. As the PM emission standard is the same for both Stage IIIB and Stage IV, it is reasonable that the two vehicles have similar PM emission factors. The decrease of the EC emission factor during the day with HVO30 may therefore have been caused by the fuel change and not the difference between the two vehicles. However, for NO_x_, the Stage IV loader has a selective catalytic reduction system, which should reduce the NO_x_ emission. This is seen as a lower NO_x_ emission factor during the day with both the vehicles in use, and the increase in the NO_x_ emission factor during the day with HVO30 is therefore likely caused by the vehicle difference and not the fuel change (Fig. 6c).

Multiple studies have shown the potential of PM and EC reduction by substituting petroleum diesel with renewable HVO, while the NO_x_ emissions are generally less affected (Bugarski et al. 2016; Zubel et al. 2016; Dimitriadis et al. 2018; McCaffery et al. 2020; Gren et al. 2021). Hence, it would be plausible to expect a similar decrease in such emissions if a full substitution were to be made, which in turn would lead to lower EC exposure concentrations, especially for the workers operating the underground loaders with lower emission standards (Stage IIIB). Saariskoski et al. (2018) investigated the emissions after a fuel change to HVO in an underground mine, but found no difference in PM (PM0.5, BC, organic matter) compared to petroleum diesel. However, this was attributed to the high use of vehicles with advanced aftertreatment (Euro V trucks with DPFs) for which PM mass emissions would be less affected by a fuel change.

#### Real-world emission factors

Even though studies of underground ambient air concentrations are important in order to assess the possible current exposure levels and the effects of renewing the vehicle fleet (Bugarski et al. 2009; Saarikoski et al. 2019), the air concentrations are heavily location dependent. For example, the efficiency of the ventilation system (air exchange rate), the volume in the section of the mine, and the number of vehicles have a high impact on the resulting air concentrations. By converting ambient air concentrations in the mines and other occupational settings to pollutant concentrations per kg burned fuel (real-world emission factors, Fig. 6c) as in this study, air concentrations can be assessed based on the vehicle types and amount of burned fuel. We suggest that this can be of important use in occupational risk assessments. No underground emission factors have previously been published, but compared to a previous study of on-road emission factors of heavy-duty diesel vehicles in an urban area, the emission factors were comparable (Larson et al. 2017). The emission factors were similar for NO_x_; 3–17 in the mine compared to 18 g kg fuel^−1^ for the heavy-duty vehicles (Larson et al. 2017), but significantly lower for PN; 9 × 10^13^ – 2.6 × 10^14^ (soot mode) in the mine compared to 4.3 × 10^15^ particles kg fuel^−1^ (particles > 50 nm). The EC emission factors in the mine (0.03–0.1 g kg fuel^−1^) were lower than the reported eBC emission (0.4 g kg fuel^−1^) for heavy-duty vehicles, but more similar if the eBC concentration showed a factor 3 higher concentration than EC as in our study. In order to better compare emissions between mines and other occupational areas, we suggest including CO_2_ measurements so that emission factors can be calculated in future studies. It should be noted that for these estimations we assume that all emissions origin from the combustion, thus non-exhaust emissions might bias the result. This is especially important for the newer vehicles that have an exhaust particle reduction unit such as a DPF. In these vehicles, the vehicle non-exhaust emission such as tire, brake and road wear might be a significant contributor to the total PM emissions.

### PAH metabolites and effect biomarkers

#### Urinary PAHs as biomarker of exposure

The miners had higher concentrations (median values) of urinary metabolites of phenanthrene and pyrene compared to values typically found in the general population in Sweden (Alhamdow et al. 2021). The urinary half-life of PAH metabolites generally are described as fast < 15 h (Li et al. 2010, 2012; Liljedahl et al. 2021), thus a urine sample after the work shift would reflect the recent day exposure. This suggests that the urinary PAH metabolites in the 27 miners here may have resulted from their occupational exposure, even though we could not find any correlation in the group of 10 miners in which both the personal airborne exposure concentrations and urinary metabolites were measured. This could be due to the limited number of subjects, other exposure routes like dermal uptake (Wahlberg et al. 2019), or as a consequence of PAH exposure from non-occupational activities. It should also be noted that the excretion times are within a couple of hours, which further indicates the occupational exposure as a likely source. However, previous publications have indicated that the correlation between airborne PAHs and metabolites are low or lacking in low-exposure scenarios (as in the range of this study) (Schoket et al. 1999; Kuusimäki et al. 2004). The correlation between airborne PAHs and metabolites have instead been found for higher exposures (around 10 times higher metabolite concentrations), such as in asphalt workers (Sobus et al. 2009). Similar levels of PAH metabolites (except of 2-Nap) as in this study have been found in garage workers occupationally exposed to diesel exhaust and firefighters (Table 3; Kuusimäki et al. 2004; Hoppe-Jones et al. 2021)). Miners in Mexico had a higher exposure of all measured PAH metabolites (Díaz de León-Martínez et al. 2021) compared to those in our study. An extensive overview of PAH metabolites in occupationally exposed workers is presented by Díaz de León-Martínez et al. (2021).

**Table 3:** Urinary metabolites in this study compared to previously published studies

Study	Population	ng/mL urine				Comments
		2-Nap	∑ OH-Flu	∑ OH-Phe	1-OH-Pyr	
Our study	Exposed miners, Sweden	3.89	0.36^a^	1.29^b^	0.31	Median conc
Alhamdow et al. (2021)	Healthy teenagers, Sweden	–	0.22^a^	0.36^b^	0.07	Median conc
Krais et al. (2021)	Short-term (3 h) exposure of diesel exhaust (PAHs 7.5 µg m^−3.^)	2.7	0.2^a^	0.4^b^	0.1	Average conc
Kuusimäki et al. (2004)	Diesel-exposed garage workers, Finland	7.18 (4.85)^g^	–	0.90 (0.61)^c,g^	0.22 (0.15)^g^	Average conc
Díaz de León-Martínez et al. (2021)	Miners, Mexico	2.54^d^	1.57^e^	3.34^f^	1.80	Median conc
Hoppe-Jones et al. (2021)	Exposed firefighters, USA	19.0^d^	0.76^e^	1.18^f^	0.24	Median conc

^a^Sum of 2- and 3-OH Flu

^b^Sum of 1,- 2-, 3- and 4-OH Phe

^c^Sum of 1,- 2-, 3-,4- and 9-OH Phe

^d^Sum of 1- and 2-Nap

^e^Sum of 2,- 3- and 9-OH Flu

^f^Sum of 1-, 2-, 4- and 9-OH Phe

^g^Original values in μmol/mol creatinine; data were transformed into density-adjusted urine using a factor of 1.48 according to Carrieri et al. (2000).

A study on short-term diesel exhaust exposure (3 h) in which healthy humans were exposed to diesel exhaust, including 7500 ng m^−3^ PAHs (16 US EPA PAHs (Wierzbicka et al. 2014)), in a chamber, did not find urinary PAH metabolites exceeding background levels (Krais et al. 2021). Even though the 8 h normalized levels (7500 × 3 h/8) would be similar to those of this study (2700 vs. 1500 ng m^−3^ of total PAHs in this study), the longer exposure time in this study (i.e., urine collected after 8 h exposure) could cause the increase in urinary PAH metabolites.

#### Urinary biomarkers of diesel toxicity

3-HPMA was found in concentrations similar to median levels reported in gasoline station workers (Frigerio et al. 2019) and a short-term exposure study with renewable diesel and petroleum diesel (Krais et al. 2021). Acrolein can be generated endogenously during lipid peroxidation and inflammation (Stevens and Maier 2008; Li et al. 2021), which can be detected in urine as 3-HPMA. However, acrolein is also known to be formed by the combustion of petroleum fuels and biodiesel, but is also present in cigarette smoke and cooked foods. 3-HPMA in our study correlated with concentrations of NO_2_, airborne exposure of phenanthrene and B[a]P, which may indicate some oxidative or inflammatory effect from the exposure. But since 3-HPMA is an unspecific marker, it is difficult to determine if it can be used as a biomarker for diesel exhaust.

4-HNE-MA, the biomarker for lipid peroxidation, was found in slightly higher concentrations than in smokers upon smoking cessation (Kuiper et al. 2010) and in similar levels compared to a short-term study with HVO exhaust (Krais et al. 2021).

The miners in this study did not show elevated urinary 8-oxodG concentrations compared to healthy volunteers in previous studies (reviewed in Barregard et al. 2013), as well as compared to short-term exposure studies with diesel (Krais et al. 2021). Exposure to combustion particles is consistently associated with oxidatively damaged DNA (Møller and Loft 2010). However, our data suggest that the exposure levels in this study were low enough to not cause measurable levels of 8-oxodG.

Although the biomarkers measured in this study were low and we did not evaluate physical symptoms, previous studies (many reviewed in Landwehr et al. 2020) have found that short-term exposure to higher concentrations of diesel exhaust (3 h, 300 µg m^−3^ PM1) may cause irritation in the upper airways (Xu et al. 2013; Wierzbicka et al. 2014). Even at shorter exposures (30–60 min, ≈ 250 to 300 µg m^−3^ PM), healthy individuals may experience irritation in the nose, throat and chest (Giles et al. 2018), impaired vascular function and reduced vasodilation (Barath et al. 2010; Lucking et al. 2011) and increased airway inflammation (Sehlstedt et al. 2010). Additionally, more recent studies with lower exposure concentrations (< 100 µg m^−3^) of petroleum diesel or renewable diesel fuels, more similar to this study, have found mild irritation symptoms (single 3 h exposure, (Gren et al. 2022)), reduced lung function and increased levels of DNA strand breaks in peripheral blood mononuclear cells (repeated 3-day exposures, (Andersen et al. 2019)). This emphasizes the importance of identifying potentially high-exposure work tasks and areas and minimizing the workers’ exposure.

Personal measurement of airborne concentrations to diesel exhaust constituents are work extensive, and the need for simpler exposure metric are needed to monitor the workers’ exposure and health. In this study we did not find any strong correlation between any of the workers airborne concentrations and the urinary PAH metabolites or biomarkers measured after the work shift. Future studies could consider evaluating a larger number of participants, include urine sampling before the work shift (in addition to after) or include other biomarkers.

### Limitations

The study was designed as an exploratory study with the aim to investigate the impact of work task, vehicle type and fuel on multipollutant personal exposure. The number of participants in each investigated group was too few to yield statistically significant power for evaluating the personal exposure toward regulatory limits. Only personnel working directly with or around diesel vehicles were included in the study, and thus only represent a part of the underground workplace. The selection of participants was also dependent on the miners to volunteer to participate in the study which potentially could have introduced some bias.

The airborne exposure was measured with multiple portable instruments including both active and passive sampling techniques. The number of pollutants that can be simultaneously measured with active sampling techniques are limited during personal measurements, as there is a physical limit on how many instruments and pumps the participants are able to carry without limiting their performance of work tasks. Passive samplers have the advantages of being small and lightweight (do not require a pump), and do not require any monitoring of flow rates during the day. Thus, passive sampling techniques (NO_2_, PAHs) enabled us to simultaneously sample a higher number of pollutants without restricting the workers in their normal work tasks, as well as to repeat the NO_2_ sampling by self-administered sampling.

We found that the ratios of PBZ/UAZ were almost identical for EC and NO_2_ across different locations in the mine. This demonstrates a high precision in both the EC and NO_2_ measurement techniques which is encouraging given that NO_2_ was measured with a passive sampler and EC with active pumped sampling. In this study we only report the sum of the US EPA 16 priority PAHs. This summed PAH concentration was > 95% in the gas phase, thus our measurements with the passive PAH sampler corresponds essentially to the gas phase only. This simplifies the interpretation of the measurements since differences in the aerosol conditions of the particle phase (amount, size and type of particles) is a factor that may affect uptake for particulate PAHs on passive samplers in a more complex way. The uptake factors for both PAH in gas and particle phase in the calibration study we conducted at UAZ were in line with published calibration studies at workplaces (Bohlin et al. 2010; Harner et al. 2013; Strandberg et al. 2018).

The passive sampling technique of NO_2_ is an accredited sampling method by IVL Swedish Environmental Research Institute, and diffusive sampling of NO_2_ is also an accepted method by NIOSH (NMAM 6700, (NIOSH 1998)). The passive sampling of PAHs has previously been evaluated and validated in various occupational settings. Nevertheless, it is important to point out that passive methods have limitations. For instance, environmental variables may influence the performance of a passive sampler (Gair and Penkett 1995; Ferm and Svanberg 1998; Strandberg et al. 2005, 2014, 2018; Bohlin et al. 2010). The levels of target compounds in the air, and interference from other chemical components, may affect the uptake in passive samplers. This is especially important for workplace air, where the concentrations of target and interfering compounds may vary significantly. High wind speed may also limit the uptake rate of compounds in passive samplers (Bohlin et al. 2010; Strandberg et al. 2014, 2018), however, lower wind speeds (< 5 m s^−1^) as in this mine should only have negligible effect on the uptake (Strandberg et al. 2005, 2018). We consider the impact of these environmental variables of the passive samplers used in this study of gaseous phase constituents to be minor but cannot rule out that they may have affected the results to some extent.

## Conclusion

The participating miners had an occupational exposure to EC and NO_2_, and for some individuals, the exposure was above or not within safe distance to the future NO_2_ and elemental carbon (EC) underground OELs. The miners also had a slightly higher urine concentration of PAH metabolites compared to the normal population. Even though it was not possible to correlate this to specific exposures, we hypothesized that it may have originated from their overall occupational activities.

We did not find that the PAH exposure was reduced by improved vehicle emission legislation. This suggest that the semi-volatile behavior of PAHs may lead to inefficient removal by the aftertreatment systems, and/or poor removal efficiency by the workplace ventilation due to gas-phase PAHs partitioning to indoor surfaces. The re-partitioning of the smaller PAHs (3–4 rings) from surfaces, such as cabin filters and clothing, are a likely cause for the increased PAH exposures compared to underground ambient concentrations. Future studies should investigate the PAH removal efficiency of aftertreatment systems in occupational settings, as well as include surface samples of PAHs to understand the contribution of secondary emissions from surfaces.

In this study, the EC exposures were lower for the operators of newer machines, potentially due to improved emission abatement techniques (with for example DPFs) compared to the older machines. Compared to previous studies in other mines or underground settings, the exposures were lower, which indicates that an increased awareness and transition to newer vehicles have contributed to an improved working environment for underground miners. This relation should be validated in future studies, as well as the impact of non-exhaust emissions and their potential health impact should be explored. In addition, in order to reduce the diesel EC exposure to below health-based limits, further improvements are needed where modern vehicles with DPFs play an important role.

## Supplementary Information

Below is the link to the electronic supplementary material.

Supplementary file1 (DOCX 838 KB)

## Data Availability

Data are available from the corresponding author on reasonable request.
