# Supplementary material for: Underground emissions and miners’ personal exposure to diesel and renewable diesel exhaust in a Swedish iron ore mine
Source: Int Arch Occup Environ Health. 2022 Mar 16;95(6):1369–88. doi: 10.1007/s00420-022-01843-x (PMC9273542; doi:10.1007/s00420-022-01843-x)
Supplement: Supplementary file 1 — Supplementary file1 (DOCX 838 KB) [file 420_2022_1843_MOESM1_ESM.docx]

### Supplementary Information

### Underground emissions and miners’ personal exposure to diesel and renewable diesel exhaust in a Swedish iron ore mine

**Louise Gren^1^, Annette M. Krais^2^, Eva Assarsson^2^, Karin Broberg^2^, Malin Engfeldt^2,3^, Christian Lindh^2^, Bo Strandberg^2,3^, Joakim Pagels^1^, Maria Hedmer^2,3*^**

^1^ Ergonomics and Aerosol Technology, Lund University, Lund, SE-221 00, Sweden
^2^ Division of Occupational and Environmental Medicine, Lund University, Lund, SE-221 00, Sweden
^3^ Department of Occupational and Environmental Medicine, Region Skåne, Lund, SE-223 81, Sweden
*Corresponding author: Maria Hedmer

**Method**

**Table S1.** Overview of the different sampling campaigns and participants. The miners included in the repeated NO_2_ sampling (including the expert NO_2_ sampling during the airborne exposure measurements) were in total 19. Participant ID no. 20-35 only participated in the biological monitoring (urine collection).

| **ID** | **Airborne exposure measurements (including expert NO_2_ sampling)** | **Biological monitoring** | **1^st^ Repeated self-administered NO_2_ sampling** | **2^nd^ Repeated self-administered NO_2_ sampling** |
| --- | --- | --- | --- | --- |
| 1 | x | x | x | x |
| 2 | x | x |  |  |
| 3 | x | x | x |  |
| 4 |  |  |  | x |
| 5 | x | x |  |  |
| 6 | x | x | x | x |
| 7 | x | x | x | x |
| 8 | x | x | x | x |
| 9 | x | x | x |  |
| 10 | x | x | x | x |
| 11 | x | x | x | x |
| 12 | x |  |  |  |
| 13 | x |  |  |  |
| 14 |  |  | x | x |
| 15 |  | x | x |  |
| 16 |  |  | x |  |
| 17 |  |  |  | x |
| 18 |  |  |  | x |
| 19 |  |  | x | x |
| 20-35 |  | x |  |  |
| Total | 12 | 27 | 12 | 11 |

#### Sampling of airborne PAHs

Airborne PAHs were determined in the personal breathing zones (PBZ) and underground ambient zones (UAZ) with a passive collection method as described in Strandberg et al. (1). The sampler was a cylindrical polyurethane foam passive air sampler (PUF-cyl, length: 10 cm, diameter: 2.2 cm) with a total surface area of 77 cm^2^ and density 0.030 g cm^-3^ (Klaus Ziemer GmbH, Germany). When used for sampling, the PUF-cyl is placed inside a protective cover net (diameter 2.2 cm, length 10 cm) with a mesh size of 1.0mm (AB Derma, Sweden). The sampler for personal measurement (PBZ) is mounted onto a holder with a support plate that can be attached using safety pins to the persons´ clothes close to the breathing zone. The samplers for the stationary measurement sites (UAZ) were placed at a height of approximately 1.5 meters above ground inside a semi-closed container mounted on a metal structure. The semi-open sampling container protects the sampler from falling dust but allows air to circulate around the sampler in a similar way to the personal (PBZ) measurement.

Before use, each PUF-cyl is pre-cleaned via Soxhlet extraction using dichloromethane for 24h, dried and stored in multiple layers of aluminium foil inside airtight Ziploc bags. Before use, and after the measurements prior to analysis, the samplers are stored in a freezer (-20°C). The PUF-cyl passive sampler has previously been evaluated and calibrated with uptake factors for short sampling times (2-8h) in other work environments (1,2). In this study, concurrent sampling with the passive method and active sampling (3) in the UAZ were performed in order to confirm that previously determined published uptake rates, are also valid in this work environment. Active sampling was performed with a flow rate of 2 L min^−1^ with a 37-mm filter mounted open-faced in conductive three-piece filter cassettes (SureSeal, SKC Inc.) collecting particle-bound PAHs followed by a solid adsorbent tube (XAD-II) collecting gas-phase PAHs.

The uptake rates obtained from the active sampling compared to the passive sampling in the UAZ were used to determine the PAH levels from the passive sampling in the PBZ. A detailed description of this calibration study will be presented separately.

*PAH analysis*

Chemicals and Reagents

All adsorbents, silica gel 60, aluminium oxide 90 active neutral and sodium sulfate (Merck, Darmstadt, Germany) were cleaned by thermal treatment at 450°C and activated at 100°C before use. All solvents were of glass distilled quality (Merck, Darmstadt, Germany). A deuterated internal standard (IS) mixture (1 ng µL^-1^) containing the 16 U.S. Environmental Protection Agency (US-EPA) priority PAHs (Dr. Ehrenstorfer (Augsburg, Germany) were used. A native mixture of all 16 priority PAHs, all at 1 ng L^-1^, (Dr. Ehrenstorfer, Augsburg, Germany), were used for detection and quantification of target compounds. Octachloronaphthalene (OCN) (Ultra Scientific, North Kingstown, RI, USA) (1 ng L^-1^) was used as recovery standard (RS).

Sample extraction, clean-up and analysis

The active (XAD-II and filter) samplers were extracted and cleaned in accordance with previously described procedures (Bramming Jorgensen et al., 2013). Each sample, filter and adsorbent were combined, spiked with 40 μL IS, and extracted in 3 mL dichloromethane using an Ultrasonic Extractor (Soltec, Italy). Final purification was done with a Pasteur pipette containing silica gel.

The PUF samples were extracted using a Dionex ASE 350 Accelerated Solvent Extractor equipment (Thermo Fisher Scientific, Inc. MA, USA). PUFs were placed in extraction cells of 60 mL filled with 4 g of silica in the bottom. Samples were spiked with 40 μL of the IS and then extracted using dichloromethane as solvent. The extraction was performed at 100°C and three static time cycles. Following extraction, all samples were evaporated and transferred to 10 mL amber vials. The samples were then concentrated under nitrogen flow until only a third of the initial volumes was left, and solvent exchanged using n-hexane (ca 3 mL), and finally evaporated to ca 1 mL. The PUFs were finally purified on a column (ID 0.9 cm) with, from top 1 g sodium sulfate, 4 g activated silica, and 2 g activated alumina. The elute was evaporated under nitrogen flow until only a third of the initial volumes was left, solvent exchanged using n-hexane (ca 3 mL), and finally evaporated to ca 200 µL. Samples were transferred to GC glass insert vials (Agilent Technologies) and 40 µL of RS was added and samples were reduced to a small volume (ca 30-40 µL) for analysis.

Target compounds were separated on an Agilent 5975C mass spectrometer (MS) coupled to a 7890A gas chromatograph (GC, Agilent Technologies). Samples (2 μL) were injected using an Agilent autosampler unit. The capillary column used was a DB-5MS (30 m × 0.25 mm, 0.25 μm, Agilent Technologies). Helium was the carrier gas at a flow rate of 1.0 mL/min. The temperature program was as follows: initial temperature 50°C for 3 min; ramp at 10°C/min to 180°C and held for 5 min; ramp at 3°C/min to 300°C and held for 20 min; injection at oven temperature at 250°C, and transfer line at 250°C. Electron impact ionization (EI) was performed at 70 eV energy and at a 230°C ion source temperature.

Pleasant recovery (65-110%) was achieved for IS standard compounds that were added to and used for correction of the samples.

Field blanks, three for each sampler type, were analyzed simultaneously with the samples. Some minor residues of some 2-4 ringed PAHs occurred but all samples were corrected for those blank values, respectively.

The limit of detection were calculated as three times the standard deviation of the values for sampler blanks or the background noise of these blanks. Moreover, a certified reference material (SRM 1649a urban dust) was used for quality control. The measured levels were considered acceptable and were for the most part within 20% of the certified levels. Good precision was realized for all duplicate results, both active and passive methods for both gaseous and particulate PAHs (precision <10-30%).

#### Analysis of urinary biomarkers

Urine samples were analyzed for PAH metabolites, namely 2-naphthol (2-Nap); 2,3-hydroxyfluorene (2,3-OH-Flu); 2,3-hydroxyphenanthrene (2,3-OH-Phe); 1-hydroxyphenanthrene (1-OH-Phe); 4-hydroxy-phenanthrene (4-OH-Phe); and 1-hydroxypyrene (1-OH-Pyr). Additionally, we analyzed 3-hydroxypropyl mercapturic acid (3-HPMA), a marker for acrolein; 4-hydroxynonenal mercapturic acid (4-HNE-MA), a marker for lipid peroxidation; and 8-oxo-2’-deoxyguanosine (8-oxodG), a marker for DNA damage, according to the method previously reported in Krais et al. (5). Briefly, 0.2 mL of urine, 0.1 mL of ammonium acetate (pH 6.5) and 0.01 mL of β-glucuronidase (*Escherichia coli*) were incubated for 30 min at 37°C. Afterwards, 0.025 mL of a 50:50 (v/v) water/acetonitrile solution and 0.025 mL of internal standards were added (final concentrations 5 ng/ml).

The plates were centrifuged for 10 min at 3,000 rpm prior to analysis.

For PAH metabolites analysis, a Luna C18(2) HST column (2.5 µm, 100 Å, 2.1 mm i.d. x 100 mm, Phenomenex, Torrance, CA, U.S.A.) was used for separation. The mobile phases were water (A) and acetonitrile/methanol (50:50, B). For 3-HPMA, 4-HNE-MA and 8-oxodG, a Restek Ulta AQ C18 column (3 µm, 4.6 mm i.d. × 100 mm, Restek, Philadelphia, PA, U.S.A.) was used for separation. Solvents were Milli-Q water (A) and acetonitrile (B), containing 0.1% formic acid.

The samples were analyzed using liquid chromatography (Shimadzu Corporation, Kyoto, Japan) coupled to a QTRAP5500 (triple quadrupole linear ion trap mass spectrometer) equipped with a TurboIon Spray source (LC-MS/MS; AB Sciex, Framingham, MA, U.S.A.).

**Results**

**Table S2.** Summary of the underground ambient and personal exposure concentrations of diesel exhaust. Both gas- and particle-phase components are included. All values are average values that were measured during the part of the work shift spent underground (GM: 7.5 hours) if nothing else is stated. Known emission standards are stated. Note that the sub-groups averages are based on a small number of measurements, but can be seen as estimates of this particular workplace.

| **ID.** | **Measurement site** | **Work task** | **Vehicle** | **Day** | **NO_2_ (µg/m^3^)** | **EC (µg/m^3^)** | **NO_2_/EC ratio** | **eBC ± Std. Dev. (µg/m^3^)** | **eBC Max. (µg/m^3^)** | **BaP (ng/m^3^)** | **16 U.S. EPA PAHs (ng/m^3^)** | **Total dust ^a^**  **(µg/m^3^)** |
| --- | --- | --- | --- | --- | --- | --- | --- | --- | --- | --- | --- | --- |
| 1 | Underground  tunnels | Tunnel service | Truck (Euro VI) | 1 | 130 | 5 | 26 | 14 ± 33 | 342 | 1.0 | 2618 | n/a |
| 2 |  | Tunnel service | Van (Euro 5) | 1 | 160 | 7 | 23 | 12 ± 16 | 142 | 0.43 | 5077 | n/a |
| 3 |  | Tunnel service | Pickup/LCV (Euro 6) | 1 | 1200 | 94 | 13 | 155 ± 165 | 670 | 0.39 | 1875 | n/a |
| - |  | Underground ambient  (roadside, -1070m) | | 1 | 760 | 41 | 19 | 112 ± 53 | 225 | <0.35 | 868 ± 14 | 565 |
| 5 | Drift 1 | Production loading | Underground loader  (Stage IIIB) | 2 | 230 | 23 | 10 | 49 ± 53 | 366 | 0.55 | 1491 | n/a |
| 6 |  | Production loading | Underground loader  (Stage IV) | 2 | 190 | 14 | 14 | 35 ± 37 | 342 | 0.45 | 2701 | n/a |
| - |  | Underground ambient | | 2 | 270 | 26 | 10 | 81 ± 32 | 134 | <0.35 | 1659 ± 48 | 401 |
| 5 | Drift 1 (HVO30) | Production loading | Underground loader  (Stage IIIB) | 3 | 340 | 16 | 21 | 38 ± 39 | 180 | <0.35 | 1279 | n/a |
| 6 |  | Production loading | - ^b^ | 3 | 63 | 0.3 | 210 | 4 ± 9 | 73 | <0.35 | 829 | n/a |
| - |  | Underground ambient | | 3 | 300 | 15 | 20 | 43 ± 23 | 92 | <0.35 | 532 ± 23 | 864 |
| 10 | Drift 2 | Production transport | Truck (Euro VI) | 4 | 150 | 8 | 19 | 24 ± 25 | 158 | <0.35 | 789 | n/a |
| 11 |  | Production loading | Underground loader  (Stage V) | 4 | 110 | <0,5 | 440 | 5 ± 20 | 315 | 0.38 | 4027 | n/a |
| 12 |  | Production transport | Truck (Euro VI) | 4 | 200 | 4 | 50 | 26 ± 29 | 214 | <0.35 | 1371 | n/a |
| 13 |  | Production transport | Truck (Euro VI) | 4 | 120 | 2 | 60 | 14 ± 24 | 248 | <0.35 | 706 | n/a |
| - |  | Underground ambient | | 4 | 86 | 3 | 29 | 16 ± 12 | 75 | <0.35 | 309 ± 35 | 1275 |
| 7 | Drift 3 | Production loading | Underground loader  (Stage IV) | 2 | 48 | 3 | 16 | 8 ± 10 | 133 | <0.35 | 510 | n/a |
|  |  |  |  | 3 | 62 | 5 | 12 | 14 ± 10 | 44 | <0.35 | 1214 | n/a |
| 8 | Drift 4 | Production loading | Underground loader  (Stage IV) | 2 | 43 | 6 | 7 | 17 ± 17 | 104 | <0.35 | 557 | n/a |
|  |  |  |  | 3 | 58 | 7 | 8 | 18 ± 16 | 68 | <0.35 | 5983 | n/a |
| 9 | Drift 5 | Production loading | Underground loader  (Stage IIIB) | 2 | 130 | 14 | 9 | N/A | N/A | <0.35 | 1428 | n/a |
|  |  |  |  | 3 | 140 | 19 | 7 | 42 ± 36 | 168^‡^ | <0.35 | 1121 | n/a |
| - | Vehicle emission test facility ^c^ | Ambient (ground level) | | 5 | 310 | 3 | 103 | 13 ± 10 | 136 | <0.35 | 291 ± 14 | 254 |
|  | Geometric mean [geometric standard deviation] | | |  |  |  |  |  |  |  |  |  |
|  | ***All underground personnel**** | | |  | *153 [2.1]* | *7 [3.7]* | *27 [3.0]* | *22 [2.3]* | *221 [1.8]* | *<0.35* | *1790 [1.9]* |  |
|  | ***Tunnel service (n=3)*** | | |  | *292* | *15* | *20* | *30* | *319* | *0.55* | *2920* |  |
|  | ***Stage IIIB & IV loader operators (n=5)*** | | |  | *110* | *11* | *10* | *27* | *174* | *<0.35* | *1700* |  |
|  | ***Stage V loader & Euro VI truck operators (n=4)*** | | |  | *141* | *2* | *71* | *14* | *227* | *<0.35* | *1320* |  |
|  | ***EU Occupational Exposure Limits Underground (current / from 2023 / from 2026)***** | | |  | ***2000/960 ^d^*** | ***-/-/50 ^e^*** | ***-*** | ***-*** | ***-*** | ***2000 ^f^*** | ***-*** |  |
| ^a^ Open-face sampling of airborne dust with a 37 mm cassette, no cut-off size available. ^b^ Worker sits in break room underground due to vehicle malfunction. ^c^ Aerosol concentrations were measured at a ground level facility. ^‡^ Data only averaged over 3.5 hours due to instrument malfunction. ^d^ 960 µg m^-3^ will be enforced at underground and tunnel workplaces from 21^st^ August 2023. ^e^ Enforced in general workplaces from 21^st^ February 2023, enforced at underground and tunnel workplaces from 21^st^ February 2026. ^f^ Swedish regulation, no EU OEL directive. * Workers in drift 1, day 3 are excluded from the GM as they were not following normal operation (id: 5 drove on HVO30 as a test, and id. 6 sat in a ventilated break room due to vehicle malfunction). ** Swedish OELs are harmonized with EU directives (6–8). | | | | | | | | | | | | |

Table S3. The median exposure concentrations (n=12) of the 16 U.S. EPA PAHs. The PAHs were measured with a passive sampling technique in the personal breathing zones (PBZ).

| **Compound** | **Median concentration (ng m^-3^)** | **Min (ng m^-3^)** | **Max (ng m^-3^)** |
| --- | --- | --- | --- |
| Naphthalene | 816 | 267 | 5263 |
| Acenaphthylene | 25 | 69 | 508 |
| Acenaphthene | 262 | 87 | 524 |
| Fluorene | 115 | 63 | 667 |
| Phenanthrene | 82 | 29 | 120 |
| Anthracene | 2.78 | 8 | 103 |
| Fluoranthene | 4.37 | 95 | 1831 |
| Pyrene | 4.79 | 4 | 34 |
| Benzo(a)anthracene | 0.18 | 46 | 828 |
| Chrysene | 0.46 | 2 | 8 |
| Benzo(b)fluoranthene | 0.60 | 35 | 1043 |
| Benzo(k)fluoranthene | 0.82 | 1 | 9 |
| Benzo(a)pyrene | 0.50 | 3 | 10 |
| Indeno(1,2,3-c,d)pyrene | 0.48 | 5 | 5 |
| Dibenzo(a,h)anthracene | <0.2 | 3 | 10 |
| Benzo(g,h,i)perylene | 0.99 | 4 | 25 |

**Table S4:** Mean values of density-adjusted urinary metabolites of PAHs and biomarkers from 27 occupationally exposed miners. The individual PAH exposures measured in the PBZ are included for 12 miners. Worker ID no. 4, 14, 16-19 are not included in this table as they only participated in the self-administered NO_2_ sampling (Fig. 2)

|  | **PAH metabolites (ng ml^-1^)*** | | | | **PAH exposure (ng m^-3^)** | | | | **Biomarkers (ng ml^-1^)** | | |
| --- | --- | --- | --- | --- | --- | --- | --- | --- | --- | --- | --- |
| **ID.** | 2-Nap | ΣOH-Flu^a^ | ΣOH-Phe^b^ | 1 OH-Pyr | Naphtalene | Fluorene | Phenanthrene | Pyrene | 4-HNE-MA | 8-oxodG | 3-HPMA |
| 1 | 5.18 | 0.35 | 1.09 | 0.29 | 1780 | 190 | 180 | 8 | 140 | 6.2 | 830 |
| 2 | 7.31 | 1.01 | 1.56 | 0.22 | 1220 | 830 | 1040 | 40 | 72 | 5.6 | 3170 |
| 3 | 7.74 | n.d. | 1.07 | 0.28 | 690 | 270 | 150 | 11 | 114 | 9.1 | 1800 |
| 5 | 5.18 | 0.41 | 2.6 | 0.68 | 940 | 120 | 90 | 6 | 104 | 11.9 | 1160 |
| 6 | 3.64 | 0.99 | 1.65 | 0.26 | 390 | 100 | 70 | 1 | 203 | 7.3 | 440 |
| 7 | 0.84 | 0.36 | 1.17 | 0.36 | 530 | 60 | 40 | 2 | 23 | 8.1 | 230 |
| 8 | 2.6 | 0.37 | 1.29 | 0.38 | 5260 | 200 | 140 | 9 | 135 | 5.1 | 220 |
| 9 | 1.78 | 0.47 | 0.89 | 0.27 | 780 | 190 | 160 | 5 | 63 | 4.6 | 520 |
| 10 | n.d. | n.d. | 2.48 | 0.84 | 480 | 70 | 40 | 1 | 4 | 4.6 | 190 |
| 11 | 7.69 | 2.52 | 6.97 | 4.02 | 3580 | 110 | 70 | 7 | 10 | 6.7 | 310 |
| 12 | N/A | N/A | N/A | N/A | 890 | 110 | 60 | 7 | N/A | N/A | N/A |
| 13 | N/A | N/A | N/A | N/A | 460 | 50 | 40 | 3 | N/A | N/A | N/A |
| 15 | 0.77 | 0.87 | 1.54 | 0.48 | N/A | N/A | N/A | N/A | 108 | 8.2 | 790 |
| 20 | 4.14 | 0.1 | 1.66 | 0.47 | N/A | N/A | N/A | N/A | 21 | 7.3 | 820 |
| 21 | 4.77 | 0.25 | 1.65 | 0.5 | N/A | N/A | N/A | N/A | 186 | 3.8 | 690 |
| 22 | 0.92 | 0.21 | 1.22 | 0.27 | N/A | N/A | N/A | N/A | 226 | 8.5 | 1740 |
| 23 | 4.93 | 0.35 | 0.98 | 0.22 | N/A | N/A | N/A | N/A | 83 | 10.6 | 450 |
| 24 | 1.81 | 0.31 | 0.96 | 0.3 | N/A | N/A | N/A | N/A | 170 | 6.8 | 1150 |
| 25 | 1.87 | 0.9 | 1.29 | 0.27 | N/A | N/A | N/A | N/A | 40 | 3.4 | 630 |
| 26 | 3.44 | 0.3 | 1.28 | 0.37 | N/A | N/A | N/A | N/A | 58 | 7.8 | 640 |
| 27 | 8.71 | 0.25 | 1.11 | 0.24 | N/A | N/A | N/A | N/A | 30 | 11.3 | 2530 |
| 28 | 2.16 | 0.46 | 0.93 | 0.24 | N/A | N/A | N/A | N/A | 57 | 8.5 | 800 |
| 29 | 8.9 | 0.29 | 0.89 | 0.25 | N/A | N/A | N/A | N/A | 40 | 6.5 | 1030 |
| 30 | 7.74 | 0.58 | 1.58 | 0.31 | N/A | N/A | N/A | N/A | 173 | 7.7 | 600 |
| 31 | 7.48 | 0.7 | 1.77 | 0.43 | N/A | N/A | N/A | N/A | 85 | 14.7 | 2960 |
| 32 | 0.22 | 0.4 | 1.18 | 0.35 | N/A | N/A | N/A | N/A | 12 | 4.9 | 210 |
| 33 | n.d. | 0.36 | 1.96 | 0.64 | N/A | N/A | N/A | N/A | 104 | 14.5 | 510 |
| 34 | 1.07 | 0.15 | 1.21 | 0.3 | N/A | N/A | N/A | N/A | 52 | 6.7 | 840 |
| 35 | n.d. | 1.21 | 2.38 | 0.59 | N/A | N/A | N/A | N/A | 91 | 18.3 | 1700 |
| * The urinary metabolites are adjusted for urine density.  ^a^ Sum of 2- and 3- OH Fluorene ^b^ Sum of Σ2,3-OH Phenanthrene, 1-OH Phenanthrene and 4-OH Phenanthrene | | | | | | | | | | | |

*
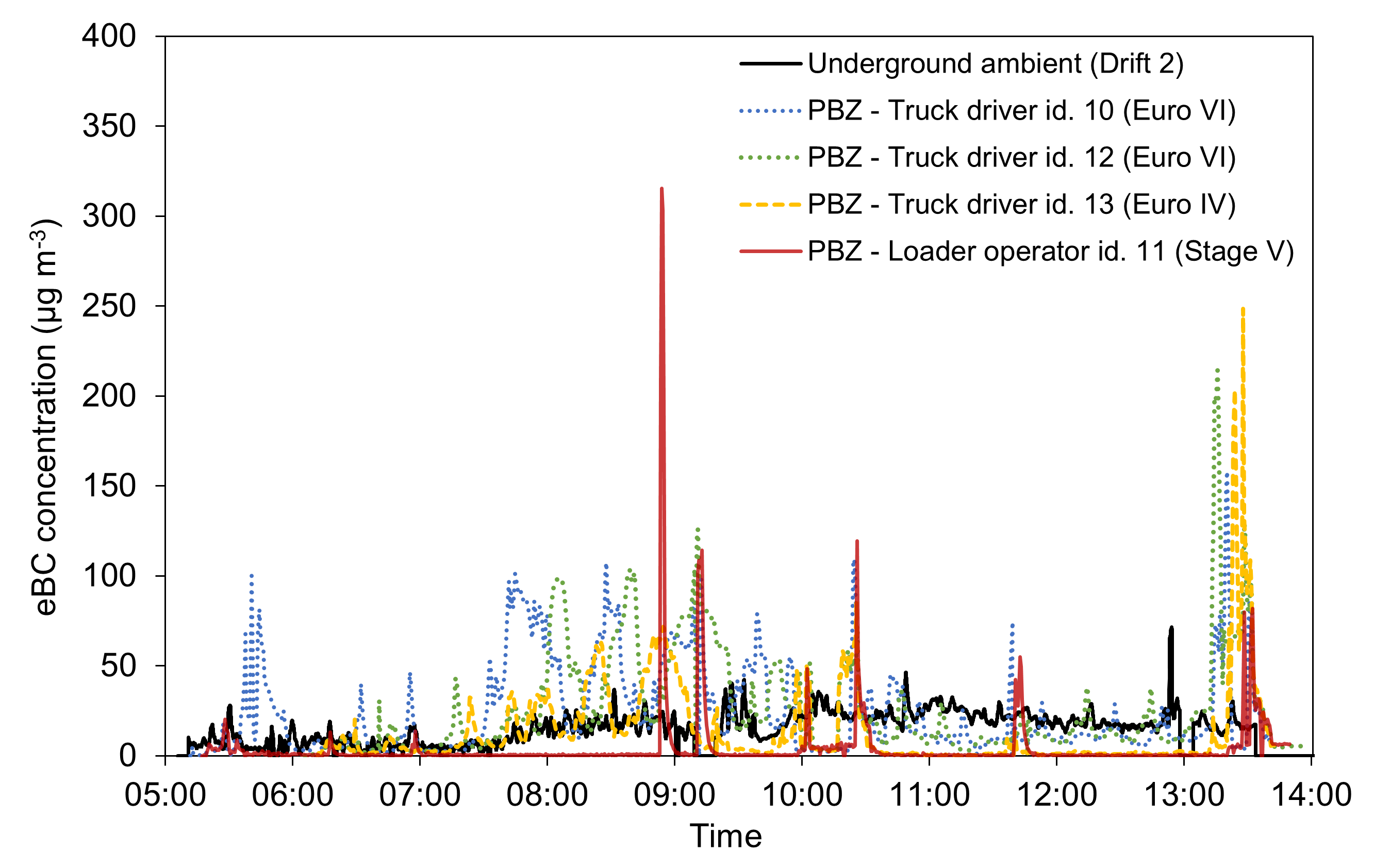
*

Figure S1. The personal exposure to eBC in Drift 2 is shown together with the measurement in the underground ambient zone (UAZ). In Dift 2 a single loader (Stage V) was operated to load the ore from the drift onto three trucks (Euro VI) for transportation to the shaft, which was located outside the drift. The concentration in the ambient zone is given with 1s resolution (shaded areas) and 1 min moving average. The PBZ is measured with 30 s resolution.

*
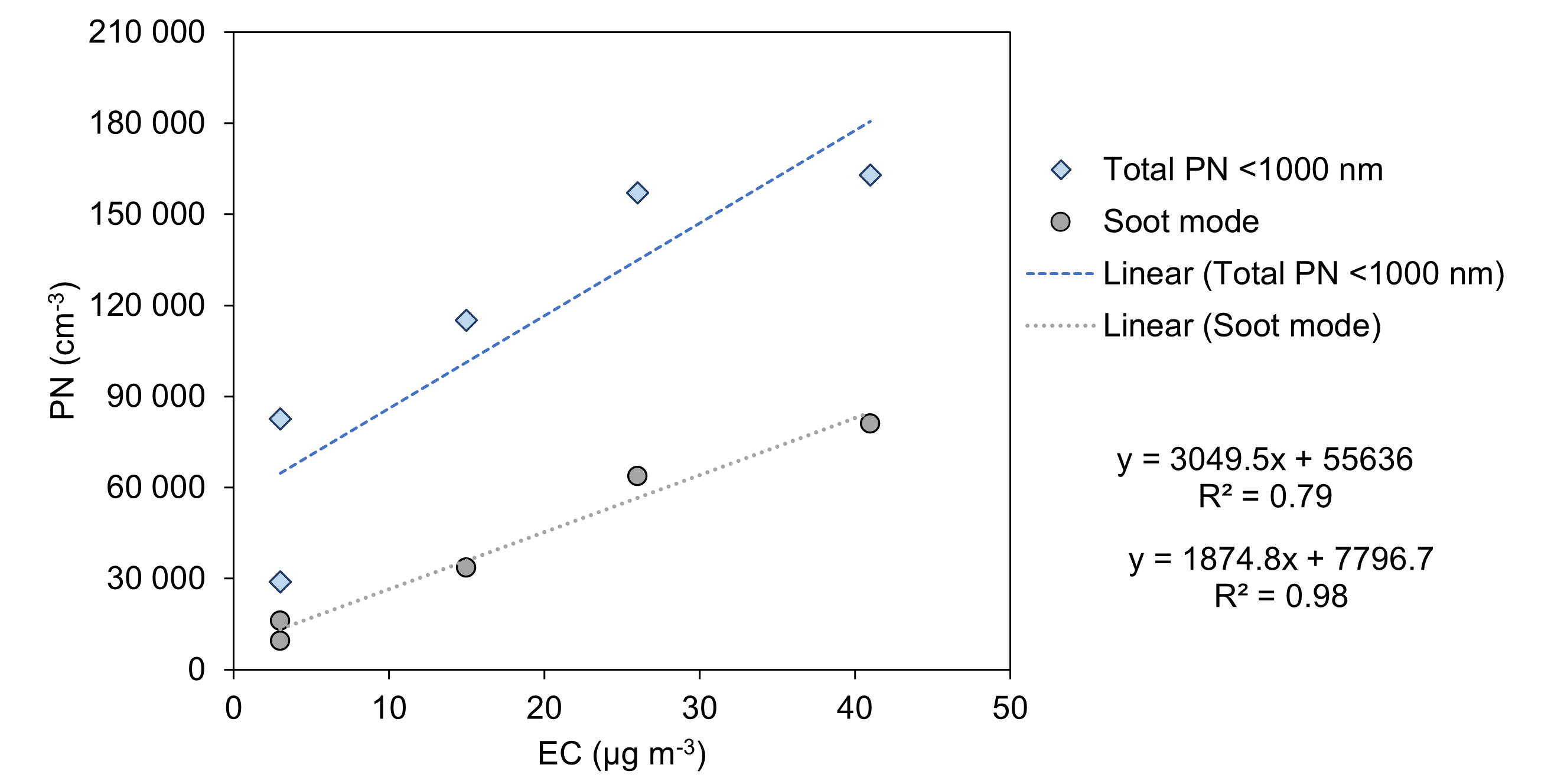
*

Figure S2. The correlation between total particle number concentration (PN), soot mode concentration, and the EC concentration in the underground ambient zones (UAZ). The correlation of EC and the soot mode concentration in the ambient zones was stronger (Pearson R=0.99, p= 0.002) than for EC and total PN (R=0.89, p=0.043), indicating that the soot mode is dominated by EC particles. This means we can use the soot mode concentration as an approximate for the solid PN concentration (as in Fig. 4c), rather than the total PN concentration which includes the semi-volatile nucleation mode particles.


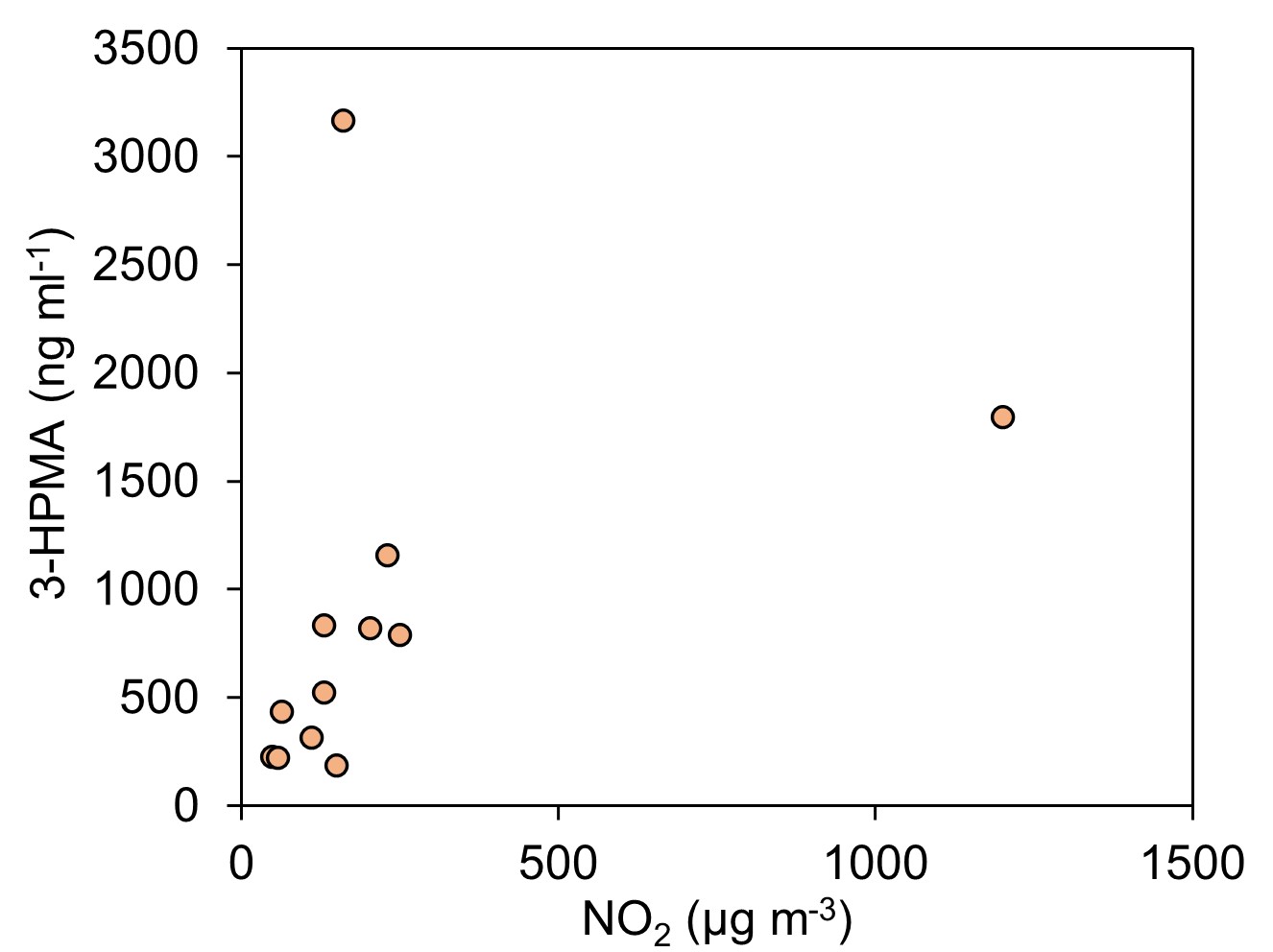

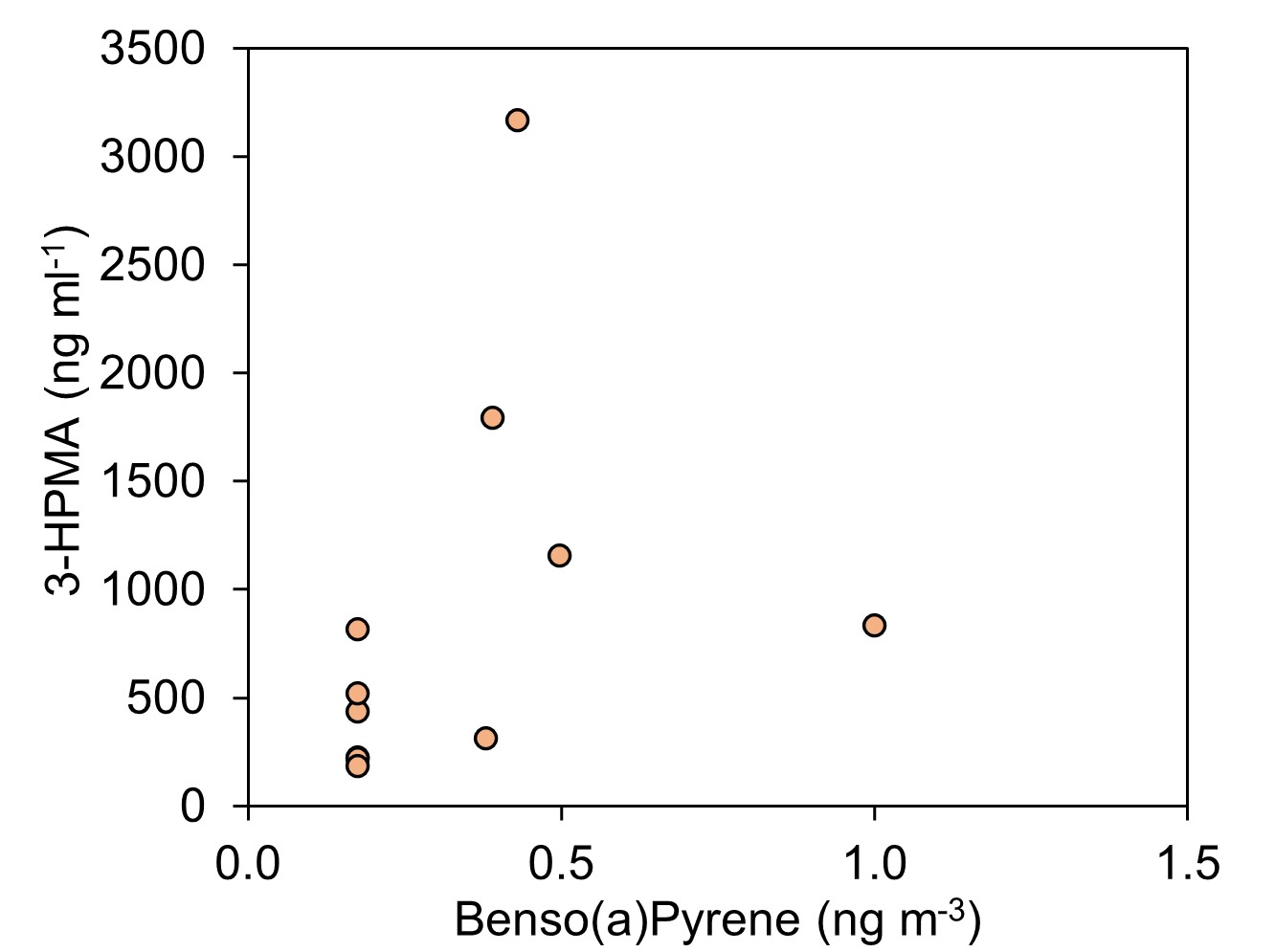


d)

c)

b)

a)


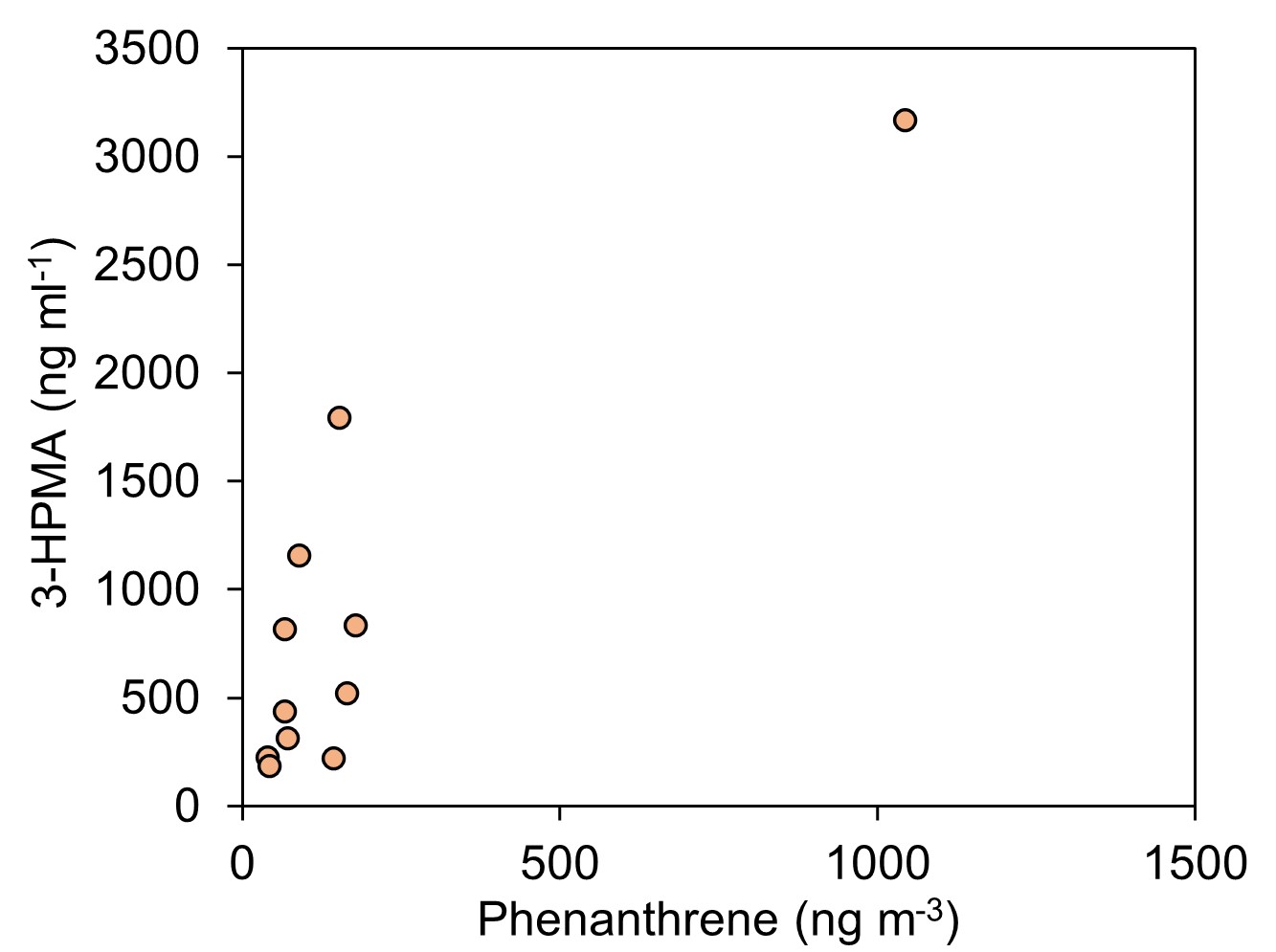

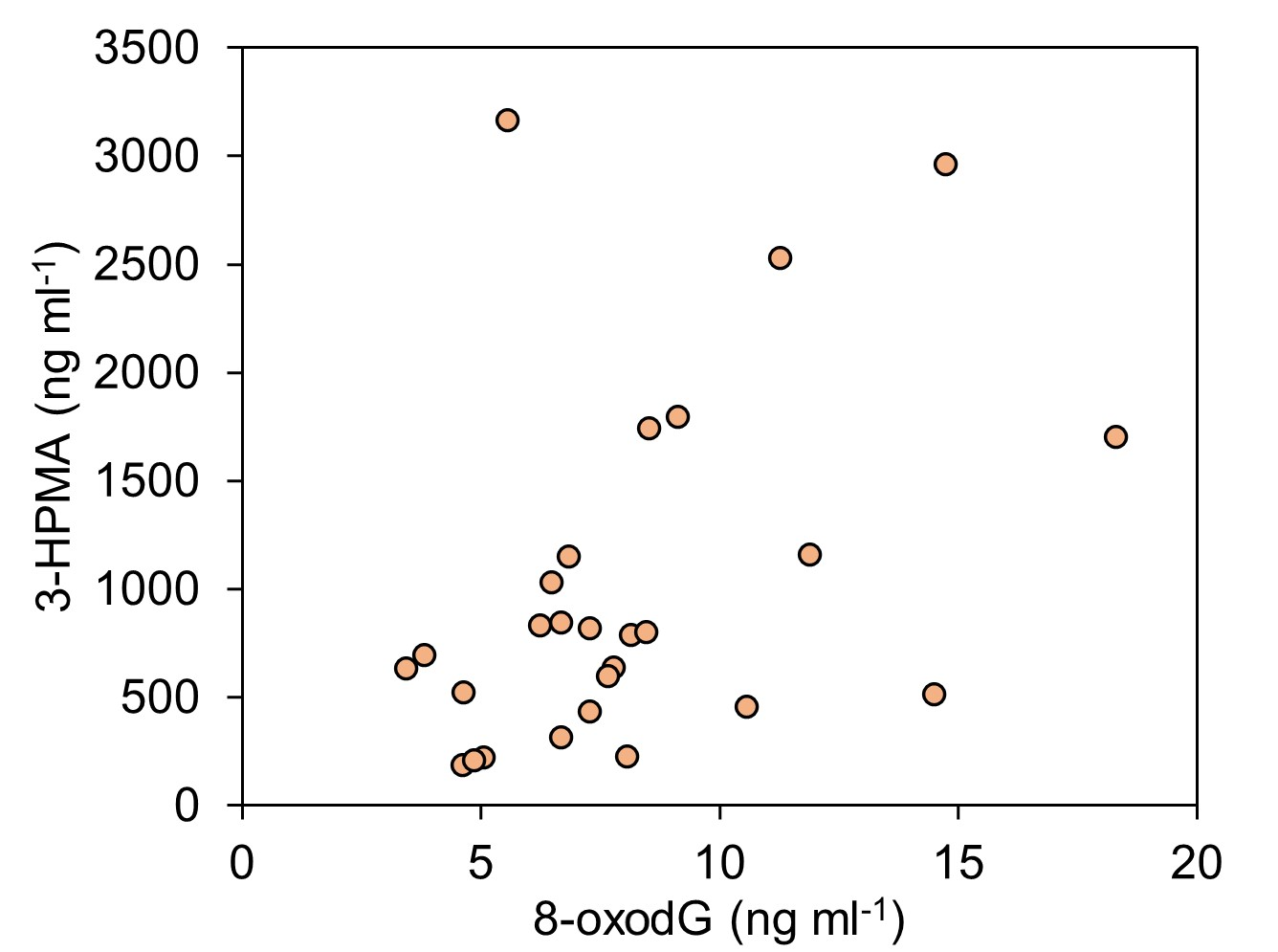


Figure S3. Correlations of 3-HPMA in urine (ng ml^-1^) and exposure concentration (m^-3^) of a) NO_2_ (Spearman’s R (R_S)_=0.681, p=0.03), b) Benso(a)Pyrene (R_S_=0.743, p=0.014), and c) Phenanthrene (R_S_=0.721, p=0.019). In d) the urinary concentration of 3-HPMA is correlated to the urinary concentration of 8-oxodG (R_S_=0.427, p=0.026).

**References**

1. Strandberg B, Julander A, Sjöström M, Lewné M, Koca Akdeva H, Bigert C. Evaluation of polyurethane foam passive air sampler (PUF) as a tool for occupational PAH measurements. Chemosphere. 2018;190:35–42.

2. Bohlin P, Jones KC, Levin J-O, Lindahl R, Strandberg B. Field evaluation of a passive personal air sampler for screening of PAH exposure in workplaces. J Environ Monit. 2010;12(7):1437.

3. NIOSH. Polynuclear aromatic hydrocarbons by GC - Method 5515. NIOSH Man Anal Methods. 1994;5506(2):1–7.

4. Jørgensen RB, Strandberg B, Sjaastad AK, Johansen A, Svendsen K. Simulated restaurant cook exposure to emissions of PAHs, mutagenic aldehydes, and particles from frying bacon. J Occup Environ Hyg. 2013;10(3):122–31.

5. Krais AM, Essig JY, Gren L, Vogs C, Assarsson E, Dierschke K, et al. Biomarkers after controlled inhalation exposure to exhaust from hydrogenated vegetable oil (HVO). Int J Environ Res Public Health. 2021;18(12):6492.

6. Directive (EU) 2019/130. The protection of workers from the risks related to exposure to carcinogens or mutagens at work. European Parliament, Council of the European Union. 2019. Available from: http://data.europa.eu/eli/dir/2019/130/oj

7. Directive (EU) 2004/37/EC. The Protection of Workers from the Risks Related to Exposure to Carcinogenic or Mutagens at Work. European Parliment, Council of the European Union. [Internet]. 2004. Available from: http://data.europa.eu/eli/dir/2004/37/2019-07-26

8. Swedish Work Environment Authority. AFS 2020:6, Occupational exposure limits. 2020.
